# Addition of Phentermine‐Topiramate to a Digitally Enhanced Lifestyle Intervention: A Double‐Blind Randomized Clinical Trial

**DOI:** 10.1002/oby.70108

**Published:** 2026-01-21

**Authors:** Alejandro Campos, Wissam Ghusn, Lizeth Cifuentes, Daniel Sacoto, Sima Fansa, Diego Anazco, Maria L. Ricardo‐Silgado, Anas Hashem, Megan Schaefer, William S. Harmsen, Heather J. Gunn, Craig Peterson, Deborah Larsen, Santosh T. Varghese, Maria D. Hurtado, Andres Acosta

**Affiliations:** ^1^ Precision Medicine for Obesity Program, Division of Gastroenterology and Hepatology, Department of Medicine Mayo Clinic Rochester Minnesota USA; ^2^ Division of Biomedical Statistics & Informatics, Department of Health Sciences Research Mayo Clinic Rochester Minnesota USA; ^3^ Vivus LLC Campbell California USA; ^4^ Division of Endocrinology, Diabetes and Metabolism, Department of Medicine Mayo Clinic Jacksonville Florida USA

**Keywords:** digitally enhanced lifestyle intervention, obesity medication, phentermine‐topiramate, telehealth, wearable technology

## Abstract

**Objective:**

This study compared the effects of phentermine‐topiramate‐ER (mid‐dose 7.5/46 mg) versus placebo on weight loss and cardiovascular disease (CVD) risk outcomes when used as an adjunct to a digitally enhanced lifestyle intervention (DELI).

**Methods:**

We conducted a 12‐month, randomized, double‐blind, placebo‐controlled trial at a single tertiary academic center in the United States (June 2020–June 2022). Eighty participants with obesity (BMI ≥ 30 kg/m^2^) were enrolled in the DELI program, consisting of in‐person and telehealth modalities, dietary and physical activity goals, and use of a smartphone application integrated with digital devices (Apple Watch and Bluetooth‐enabled weight scale and blood pressure monitor). Participants were randomized 1:1 to receive either phentermine‐topiramate‐ER (*n* = 42) or placebo (*n* = 38) in addition to the DELI.

**Results:**

At 3 months, the phentermine‐topiramate group lost a mean of 10.82 kg versus 4.04 kg in the placebo group (mean difference −6.78 kg; *p* = 0.002). At 12 months, weight loss was 15.32 kg versus 5.85 kg, respectively (mean difference −9.48 kg; *p* < 0.001). Participants receiving phentermine‐topiramate‐ER experienced a 3.35% reduction in the estimated atherosclerotic CVD risk compared to baseline (*p* = 0.004).

**Conclusions:**

Phentermine‐topiramate‐ER, when combined with a DELI, produced significant and sustained weight loss and reduced CVD risk in adults with obesity.

**Trial Registration:**

ClinicalTrials.gov: NCT04408586

## Study Importance

- What is already known?
  ○ The use of digital strategies—such as telehealth visits, activity trackers, and mobile applications—in weight loss interventions has increased in recent years.
  ○ Prior studies have shown modest and heterogeneous effects of these strategies on weight loss outcomes, and few have evaluated their combination with pharmacotherapy.
- What does this study add?
  ○ In adults with obesity, adding once‐daily phentermine–topiramate‐ER (mid‐dose 7.5/46 mg) to a digitally enhanced lifestyle intervention (DELI) produced significantly greater weight loss compared with placebo at 3, 6, 9, and 12 months and reduced estimated atherosclerotic cardiovascular disease risk at 12 months.
  ○ The weight loss achieved with a DELI plus phentermine–topiramate‐ER exceeded that reported in prior phentermine–topiramate trials without DELI.
- How might these results change the direction of research or the focus of clinical practice?
  ○ Incorporating a DELI into obesity management could amplify the effects of both pharmacologic and lifestyle‐only approaches while improving cardiometabolic outcomes.
  ○ Future studies should directly compare a DELI with traditional lifestyle programs, evaluate its combination with other obesity medications, and assess cost‐effectiveness and scalability across health care settings.

## 1 Introduction

Obesity is a chronic, heterogeneous, and multifactorial disease, and it is the most common noncommunicable disease worldwide [1]. By 2030, it is estimated that 1 billion of the world's population will be affected by obesity [2]. Importantly, obesity increases the risk of type 2 diabetes, hypertension, and dyslipidemia, increasing the risk of cardiovascular disease and mortality [3], representing 18% of all deaths related to preventable noncommunicable diseases [4, 5]. In the United States, obesity has an estimated annual cost of $480 billion [6].

Lifestyle interventions (e.g., dietary, behavioral, physical activity changes) continue to be the cornerstone of weight loss interventions to treat obesity; however, lifestyle intervention alone rarely induces significant and sustained weight loss [7]. Digital strategies such as smartphone applications and digital devices have been used to monitor progress, improve adherence, and enhance the effects of traditional lifestyle intervention [8, 9]. The implementation of telehealth (i.e., visits through remote video call) can improve obesity management by increasing access, facilitating follow‐up, and decreasing the burden of in‐person visits. Telehealth strategies have been shown to be promising [10], but the overall effect of digitally enhanced lifestyle intervention (DELI) programs (i.e., combination of traditional in‐person intervention with telehealth component including digital devices) in long‐term and randomized studies is modest [11, 12, 13, 14, 15, 16].

To achieve a greater weight loss response, guidelines support the addition of antiobesity medications (AOMs) to lifestyle intervention [17, 18, 19]. To the date of this publication, there are six FDA‐approved medications for the long‐term treatment of obesity. These medications have demonstrated a variable weight loss response between 6% and 16% total body weight loss (TBWL) after 12 months of therapy in randomized controlled trials [19] and real‐world cohort studies [20, 21]. Among these options, the combination of phentermine, a sympathomimetic and anorexigenic agent, with topiramate, an anticonvulsant known for its anorexigenic effects, has demonstrated a safe side effect profile while achieving an 8.1‐kg weight loss over a 56‐week period [22].

The effect of AOMs as an adjunct to a DELI on body weight in adults with obesity has not been yet studied. We hypothesized that the modest effects of previous studies using a DELI for the treatment of obesity could be enhanced by the addition of AOMs. For this reason, we designed a DELI program to maximize weight loss in adults with obesity. Here, we report the weight loss outcomes of a 12‐month, single‐center, double‐blind, and randomized trial that examined the weight loss effect of a medium dose of phentermine‐topiramate extended release (ER), compared to placebo, as an adjunct to a DELI program. The primary endpoint of this study was weight change from baseline in kg between both groups at 3 months.

## 2 Methods

### 2.1 Trial Design

This was a 12‐month, single‐center, double‐blinded, randomized trial. The study was conducted with approval from the institutional review board (IRB 19‐011697) at the Mayo Clinic, Rochester, MN. The protocol is available in the online Supporting Information. The clinical trial was registered on ClinicalTrials.gov (NCT04408586). All accrued participants signed the informed consent after a thorough explanation by a study member. Eligible participants were randomly assigned to either phentermine‐topiramate‐ER (7.5/46 mg) or placebo for 12 months. All randomized participants received a set of generic, consumer‐grade, Bluetooth low energy (BLE) Sichtec weight scale and BLE Urion blood pressure monitor devices designed for general use, along with access to a smartphone application (VitalTech LLC) to participate in a DELI and be randomized to either phentermine‐topiramate‐ER or placebo. Participants were allowed to keep all the digital devices upon completion of the study (i.e., completing the 12‐month in‐person visit).

### 2.2 Participants

We recruited participants from Rochester, MN, by advertising the study at the Mayo Clinic's classifieds website, targeted social media advertising campaigns, and the Mayo Clinic Weight Management Clinic. Initial contact with prospective participants occurred via email or telephone, during which coordinators provided study information and answered questions; ineligibility was occasionally identified at this stage prior to formal screening. Participants were eligible if they were 18 to 75 years of age, had body mass index (BMI, weight in kilograms divided by height in meters squared) > 30 kg/m^2^, owned a mobile device running iOS 13 or later, and had access to a wireless internet connection (Wi‐Fi or mobile data). Exclusion criteria included: history of bariatric procedure; weight greater than 450 lb (204 kg, due to scale limitations); use of AOMs within 3 months before study randomization; change in total body weight of > 3% within 3 months before study randomization; history of chronic gastrointestinal or systemic diseases and/or medications that could affect gastrointestinal motility, medication absorption, or appetite within 6 months before study randomization; significant untreated psychiatric condition; coronary artery disease; heart failure; history of glaucoma, history of nephrolithiasis; known allergy to phentermine or topiramate; and current or planned pregnancy. A full list of the eligibility criteria is provided in the online Supporting Information.

### 2.3 Trial Interventions

The study intervention consisted of the addition of phentermine‐topiramate‐ER or placebo to a DELI that combined a traditional in‐person lifestyle intervention with a telehealth modality and the use of digital devices integrated into a smartphone application over 12 months. Participants attended a total of 16 visits—8 in person and 8 via telehealth (videoconferencing)—as outlined in Figure S1. The smartphone application integrated digital devices to support self‐monitoring and goal adherence in addition to a video call to perform the telehealth visits. The digital devices included weight scale and blood pressure monitor devices designed for general use and a wearable activity tracker (Apple Watch Series 5 or 6). These digital devices automatically transmitted weight, blood pressure, step count, resting and exercise heart rate, and estimated daily calorie expenditure data to the study smartphone application (VitalTech) as the measurements were recorded by each device. Daily calorie expenditure was estimated using Apple's proprietary heart rate–calorimetry model, which combines personal characteristics (age, sex, weight, and height) with sensor‐derived data (heart rate and accelerometer) collected by the Apple Watch. Participants were instructed to follow standardized lifestyle goals, including a low‐calorie diet, physical activity, avoidance of liquid calories, and regular monitoring of blood pressure and weight using the provided digital devices.

The eight in‐person visits included three preintervention visits (screening, testing, and randomization visits) and five follow‐up visits at 1, 3, 6, 9, and 12 months. The eight telehealth visits occurred at 2 weeks and then at 2, 4, 5, 7, 8, 10, and 11 months post randomization. There was no fixed duration for study visits; however, in‐person visits typically lasted approximately 30 min, and telehealth visits averaged around 15 min, with slight variations among participants. All visits were completed by a study physician. In‐person study visits were conducted in the Clinical Research Trials Unit (CRTU), and telehealth visits were conducted via video call using a web‐based digital platform by the study investigators and a smartphone application (VitalTech) by the study participants. Texting functionality was enabled in the application, but participants were encouraged to use the video call mode. In addition to evaluating clinical outcomes within this trial, participant data and outcomes were also used in a separate, independently reported analysis aimed at assessing weight loss responses stratified by a machine‐learning‐assisted genetic risk score for calories‐to‐satiation (CTS_GRS_) [23].

### 2.4 Digitally Enhanced Lifestyle Intervention

We asked all participants to follow the same lifestyle intervention goals: (1) low‐calorie diet (1200 cal/day for females and 1400 cal/day for males), (2) 10,000 daily steps tracked on the provided Apple Watch device, (3) 150 min of exercise a week, (4) no liquid calories or artificially sweetened beverages, (5) daily use of the Apple Watch and daily use of the provided Bluetooth‐enabled blood pressure monitor, and (6) weekly weight measurement using the provided Bluetooth‐enabled weight scale.

During the randomization visit, participants had an in‐person encounter with a registered dietitian and a study physician. The dietitian provided detailed dietary counseling based on the study's standard lifestyle intervention approach and provided educational material to calculate or estimate calories from foods or meals to adhere to the low‐calorie goal. The study physician explained the DELI, verified the access to the smartphone application, and provided the digital devices (i.e., Apple Watch Series 5 or 6, Bluetooth‐enabled weight scale, and Bluetooth‐enabled blood pressure monitor) and the randomly assigned study medication or placebo. During the randomization and the in‐person follow‐up visits we collected blood to measure a basic metabolic panel, glycated hemoglobin (HbA1c), high sensitivity C‐reactive protein (hsCRP), and a lipid profile. A body composition analysis with dual‐energy x‐ray absorptiometry (DXA) was done at the randomization and 12‐month visits.

During each in‐person and telehealth follow‐up visit, participants were counseled on meeting lifestyle intervention goals, medication adherence, side effects, consistent use of the digital devices, data tracking, and troubleshooting, and negative pregnancy tests were verified in all women of childbearing potential. The data from the digital devices were used to give feedback on the lifestyle intervention and to assess for potential side effects.

### 2.5 Participant Randomization

The only difference between study groups was randomization to either phentermine‐topiramate‐ER (Vivus LLC) or matching placebo. Participants were randomized in a 1:1 ratio using a permuted block design with random block sizes of 4 and 6. Allocation was concealed: the study statistician generated the randomization code and provided it exclusively to the study pharmacist. The study investigators and participants were blinded to the assignment.

Participants randomized to phentermine‐topiramate‐ER received 3.75/23 mg daily for 14 days, after which the dose was escalated to a mid‐dose of 7.5/46 mg daily for the remainder of the study. Both active drug (3.75/23 mg and 7.5/46 mg phentermine‐topiramate‐ER) and the matching placebo were over‐encapsulated to ensure blinding. To match the titration schedule and avoid bias, all participants initially received a 14‐day supply, followed by another 14‐day supply. Thereafter, 3‐month supplies were provided at each in‐person visit.

### 2.6 Study Endpoints

The primary outcome was weight change from baseline in kg between both groups at 3 months. The secondary endpoints included weight change in kg at 6, 9, and 12 months; proportion of participants achieving ≥ 5%, ≥ 10%, ≥ 15%, and ≥ 20% weight loss at 12 months; improvement in adiposity‐associated comorbidities and parameters of cardiometabolic risk (prediabetes, type 2 diabetes, HbA1c, hypertension, systolic and diastolic blood pressure, dyslipidemia, lipid profile, and atherosclerotic cardiovascular disease risk [ASCVD]); change in body composition at 12 months; change in quality of life measurement at 12 months using the 12‐item Short Form Survey (SF‐12); and change in the number of daily steps, daily exercise duration, and daily calorie expenditure between both groups. Daily steps, daily physical activity time, and caloric consumption were aggregated to a weekly total or daily average over the week‐long period to smooth out day‐to‐day variation.

### 2.7 Power Calculation

The proposed group difference and the standard deviation (SD) of reduction in body weight are based on our pilot study (with liraglutide 3.0 mg vs. placebo). The SD for the overall weight change (pre‐post at 12 weeks) observed was 2.8 kg and observed weight loss in the control/placebo group was 6.1 kg [24]. Assuming an anticipated dropout rate of 10% by the 3‐month assessment, we estimated that an enrollment target of 82 participants (41 per group) would provide the trial with greater than 90% statistical power to detect a meaningful difference of 2 kg in body weight between the placebo and phentermine‐topiramate‐ER groups at 3 months, based on a two‐sample *t*‐test assuming a pooled SD of 3.0 kg (conservatively higher than 2.8 observed in the prior study) with a two‐sided alpha level of 0.05.

### 2.8 Statistical Analysis

Descriptive statistics are reported as frequency and percentage for discrete variables and as mean and SD for continuous variables. Two‐sample *t*‐tests and chi‐square tests were used to compare baseline demographics, anthropometrics, cardiometabolic parameters, and wearable characteristics between the randomization assignment of phentermine‐topiramate‐ER with DELI versus placebo with DELI. The effect of phentermine‐topiramate‐ER on primary and secondary endpoints was examined under intention‐to‐treat (ITT) principles [25]. Measurements were made at randomization and at 1, 3, 6, 9, and 12 months. A mixed‐effects model, where time and treatment and the time*treatment interaction were fixed effects, with a random intercept and slope (time) per subject, was used for handling missing data for the rest of the endpoints. This model adjusts for the correlation between the multiple measures per subject and allows use of all available measurements for each subject. An unstructured correlation was assumed in the models. Analyses used the Proc Mixed procedure in SAS, version 9.4. A per protocol principle was used to evaluate some secondary endpoints in completers of the trial. Assessment of the effect of phentermine‐topiramate‐ER was made using ANCOVA, including the baseline measurement of the outcome examined. The percentage of missing values across the six time points varied between 0% and 25%. As a sensitivity analysis, we used multiple imputation to fill in the missing weight values at 1, 3, 6, 9, and 12 months. We imputed 100 datasets using fully conditional specification with the Blimp 3.0 application [26] because it easily incorporates a multilevel data structure. The imputation model was a mixed‐effects model with body weight as the outcome and time, treatment, time*treatment, time*time, and time*time*treatment as fixed effects. Convergence of the Markov chain Monte Carlo (MCMC) algorithm was determined by calculating potential scale reduction factors (PSRF) for each parameter and examining trace plots of the parameters to evaluate mixing of the Markov chains. If all PSRF values were below 1.10, then we concluded that the model converged [27]. The burn‐in period of 5000 and between‐imputation interval of 2500 were determined by the number of iterations it took to get PSRF values below 1.10. The analysis model was identical to the imputation model. Analysis results were pooled using Rubin's rules. A two‐sided *p* value of less than 0.05 was set for statistical significance. The 10‐year and lifetime ASCVD risk scores were calculated using the ASCVD Risk Estimator Plus in a per protocol fashion for participants who had available laboratory data at baseline and at 9 or 12 months. The required parameters for ASCVD risk calculation are available at: https://tools.acc.org/ascvd‐risk‐estimator‐plus/#!/calculate/estimate/.

### 2.9 Use of Data for External Validation of Genetic Risk Score and Weight Loss Outcomes

A subset of participants from this clinical trial was included in a separate, independently reported study aimed at evaluating the clinical utility of a machine‐learning‐derived genetic risk score, known as the Calories‐to‐Satiation Genetic Risk Score (CTS_GRS_) [23]. This score was originally developed in a distinct population using deep‐phenotyping data, including ad libitum meal tests and imaging‐based gastric physiology measures. In the external validation phase, 50 participants from this trial who completed baseline phenotype testing and had available genetic data were included. The CTS_GRS_ was applied to these individuals to predict their likelihood of achieving high CTS and the external validity was assessed by evaluating its predictive performance. Furthermore, weight loss outcomes at 52 weeks were stratified by both observed CTS (derived from ad libitum meal testing) and CTS_GRS_. These analyses were conducted in a blinded fashion as detailed in the cited manuscript [23].

## 3 Results

### 3.1 Participants Characteristics

**FIGURE 1:**
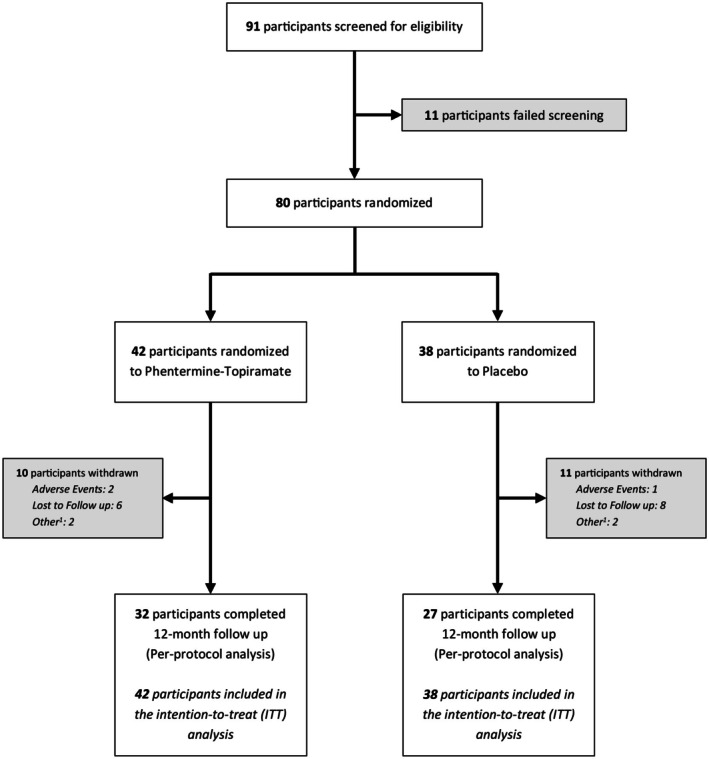
Flow diagram. ^1^Included personal or medical reasons unrelated to the study or the study medication.

The study was conducted from June 2020 to June 2022. Ninety‐one participants were assessed for eligibility. A total of 80 participants were randomized, 42 to the phentermine‐topiramate‐ER group and 38 to the placebo group (Figure 1). Overall, 90% of the participants reached the 3‐month primary endpoint. At 12 months, 32 participants in the phentermine‐topiramate‐ER group and 27 in the placebo group completed the study, representing an overall completion rate of 66%.

Demographics and clinical baseline characteristics were similar across both groups (Table 1). Participants were mostly middle‐aged with a mean age of 42.2 (10.6) years, and most were female (85%) and White (98%). The mean body weight was 109.4 (18.9) kg and BMI 42.0 (19.7) kg/m^2^. The percentage of participants with one or more obesity‐related comorbidities was 52.5%, and 23.8% of participants had prediabetes/diabetes.

**TABLE 1:** Demographic and clinical characteristics of the participants at baseline.^a^

Characteristic	Phentermine‐topiramate‐ER plus DELI (*N* = 42)	Placebo plus DELI (*N* = 38)
Age—years	43.4 ± 10.9	40.8 ± 10.2
Female sex—*n* (%)	36 (85.7)	32 (84.2)
Self‐reported White race—*n* (%)^b^	42 (100.0)	37 (97.4)
Body weight—kg	108.3 ± 17.3	110.5 ± 20.6
BMI—kg/m^2^ ^c^	40.5 ± 17.9	43.6 ± 21.6
Waist circumference—cm	114.3 ± 12.7	115.8 ± 14.7
Hip circumference—cm	126.9 ± 10.9	127.9 ± 14.9
Pulse—beats/min	73.8 ± 8.8	76.7 ± 12.2
Systolic blood pressure—mm Hg	133.0 ± 16.0	131.6 ± 13.4
Diastolic blood pressure—mm Hg	81.8 ± 10.5	81.1 ± 10.0
Fasting glucose—mg/dL	103.1 ± 34.9	97.3 ± 9.5
Glycated hemoglobin—%	5.5 ± 0.8	5.4 ± 0.5
Diabetes/prediabetes— *n* (%)	12 (28.6)	7 (18.4)
Triglycerides—mg/dL	135.6 ± 107	124.2 ± 48.8
LDL cholesterol—mg/dL	110.5 ± 27.2	110.1 ± 37.2
HDL cholesterol—mg/dL	52.1 ± 13.1	48.9 ± 13.1
hsCRP—mg/L	5.5 ± 7.7	6.3 ± 7.0
ASCVD 10 years—%^d^	2.7 ± 2.6	1.8 ± 1.5
ASCVD lifetime risk—%^d^	34.5 ± 11.9	33.0 ± 11.6
Wearable‐tracked characteristics		
Step count—steps/day	6534 ± 2993	5700 ± 2854
Resting pulse—beats/min	59.6 ± 7.2	59.3 ± 7.2
Exercise maximal pulse—beats/min	132.3 ± 13.9	128.6 ± 16.7
Daily calorie expenditurekcal^e^	1987 ± 395	1990 ± 460
Body composition^f^		
Total fat mass—kg	51.7 ± 12.2	52.9 ± 13.4
Total fat mass—%	47.7 ± 6.4	47.6 ± 5.6
Total lean mass—kg	52.9 ± 8.9	54.7 ± 9.8
Total lean mass—%	49.4 ± 6.0	49.8 ± 5.2

^a^ Plus‐minus values represent mean ± SD. DELI denotes digitally enhanced lifestyle intervention, HDL denotes high‐density lipoprotein, LDL low‐density lipoprotein, and hsCRP high sensitivity C‐reactive protein.

^b^ Race was self‐reported by participants.

^c^ BMI is calculated as the weight in kilograms divided by height in meters squared.

^d^ ASCVD scores were calculated using the ASCVD Risk Estimator Plus in a per protocol fashion for patients who had available laboratory data at baseline and at 9 or 12 months. The required parameters for ASCVD risk calculation are available at: https://tools.acc.org/ascvd‐risk‐estimator‐plus/#!/calculate/estimate/. For the 10‐year ASCVD risk score, 23 participants in the phentermine‐topiramate‐ER group and 14 participants in the placebo group had available data. For the lifetime ASCVD risk score, 34 participants in the phentermine‐topiramate‐ER group and 28 participants in the placebo group had available data.

^e^ Daily calorie expenditure were estimated using Apple's proprietary heart rate–calorimetry model, which combines personal characteristics (age, sex, weight, and height) with sensor‐derived data (heart rate and accelerometer) collected by the Apple Watch.

^f^ Body composition analysis was done by dual‐energy x‐ray absorptiometry (DXA). Baseline results are available for 40 patients in the phentermine‐topiramate‐ER group and 35 patients in the placebo group.

### 3.2 Weight Loss

**FIGURE 2:**
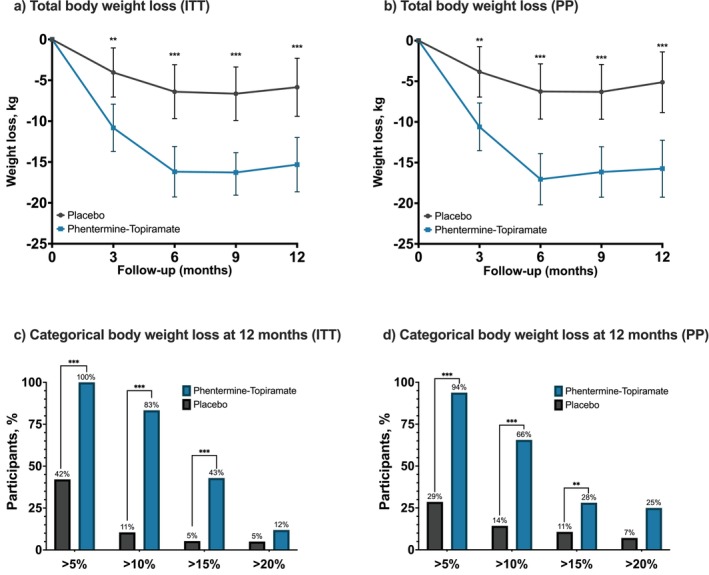
Upper panel: Total body weight loss at 3, 6, 9, and 12 months by (a) intention‐to‐treat (ITT) and (b) per protocol (PP) analysis. Lower panel: Proportion of participants achieving a categorical total body weight loss of ≥ 5%, ≥ 10%, ≥ 15%, and ≥ 20% at 12 months by (c) intention‐to‐treat (ITT) analysis and (d) per protocol (PP) analysis. **: *p* ≤ 0.01; ***: *p* ≤ 0.001. [Color figure can be viewed at wileyonlinelibrary.com]

The combination of a medium dose of phentermine‐topiramate‐ER with DELI resulted in greater weight loss response compared with DELI alone throughout the study. In the ITT analysis, the mean weight change at 3 months was −10.82 kg with phentermine‐topiramate‐ER, as compared with −4.04 kg with placebo (mean diff. −6.78 kg; 95% confidence Interval [CI], −10.95 to −2.60; *p* = 0.002). At 6 months, participants in both groups achieved their mean nadir weight loss, with 16.19 kg in the phentermine‐topiramate‐ER group compared with 6.40 kg in the placebo group (mean diff. −9.80 kg; 95% CI, −14.31 to −5.28; *p* < 0.001). At the end of the trial, participants in the phentermine‐topiramate‐ER group had a 15.32‐kg weight loss compared to 5.85 kg in the placebo group (mean diff. −9.48 kg; 95% CI, −14.34 to −4.61; *p* < 0.001) (Figure 2a).

As for per protocol analysis, the mean weight change at 3 months was −10.61 kg with phentermine‐topiramate‐ER, as compared with −3.84 kg with placebo (mean diff. −6.77 kg; 95% CI, −11.04 to −2.50; *p* = 0.003). At 6 months, participants in both groups achieved their mean nadir weight loss, with −17.05 kg in the phentermine‐topiramate‐ER group compared with −6.26 kg in the placebo group (mean diff. −10.79 kg; 95% CI, −15.42 to −6.16; *p* < 0.001). And at 12 months, participants in the phentermine‐topiramate‐ER group had a −15.74‐kg weight loss compared to −5.12 kg in the placebo group (mean diff. −10.63 kg; 95% CI, −15.73 to −5.52; *p* < 0.001) (Figure 2b). Finally, for the multiple imputation analysis, the model‐based mean weight change at 3 months was −9.04 kg with phentermine‐topiramate‐ER, as compared with −4.00 kg with placebo (mean treatment diff. −7.73 kg; 95% CI, −15.56 to 0.11; *p* = 0.053). Mean weight change at 6 months was −14.34 kg in the phentermine‐topiramate‐ER group compared with −6.00 kg in the placebo group (mean treatment diff. −11.05 kg; 95% CI, −18.83 to −3.28; *p* = 0.005). At the end of the trial, participants in the phentermine‐topiramate‐ER group had a 9.07‐kg weight loss compared to 4.04 kg in the placebo group (mean treatment diff. −12.37 kg; 95% CI, −20.34 to −4.41; *p* = 0.001).

Participants in the phentermine‐topiramate‐ER group were more likely than placebo participants to experience weight loss of 5% or more (100%; 42 participants vs. 42%; 16 participants), 10% or more (83%; 35 participants vs. 11%; 4 participants), and 15% or more (43%; 18 participants vs. 5%; 2 participants) of baseline body weight at 12 months (Figure 2c). The percentage of participants who lost more than 20% body weight was 12% in the phentermine‐topiramate‐ER group compared to 5% in the placebo group (*p* = 0.31).

### 3.3 Atherosclerotic Cardiovascular Disease Risk (ASCVD) and Metabolic Parameters

Participants in the phentermine‐topiramate‐ER group had a decrease in their lifetime ASCVD risk (−3.35%) compared to the placebo group (3.43%; difference −6.78%; 95% CI, −9.92% to −3.64%; *p* = 0.004; Figure S2). Compared to placebo, the phentermine‐topiramate‐ER group had a decrease in waist circumference (−12.6 cm with phentermine‐topiramate‐ER vs. −2.1 cm with placebo; difference −10.5 cm; 95% CI, −15.20 to −5.86), BMI (−5.07 kg/m^2^ with phentermine‐topiramate‐ER vs. −1.88 kg/m^2^ with placebo; difference −3.19 kg/m^2^; 95% CI, −4.73 to −1.65), and diastolic blood pressure (−4.79 mm Hg with phentermine‐topiramate‐ER vs. −1.16 mm Hg with placebo; difference −3.62 mm Hg; 95% CI, −7.20 to −0.05) (Table 3 and Tables S1 and S2). At 12 months, participants in the phentermine‐topiramate‐ER group had a decrease in fat mass percentage and an increase in lean mass percentage (Table 2 and Table S3). There were no statistical differences among the groups in systolic blood pressure, HDL and LDL cholesterol, triglycerides, hsCRP, HbA1c, or resting and exercise heart rate (Table 3 and Table S2). For the 10‐year ASCVD risk score and the lifetime ASCVD risk score, 34 participants in the phentermine‐topiramate‐ER group and 28 participants in the placebo group had available data.

**TABLE 2:** Primary and selected secondary endpoints.

Endpoints	Phentermine‐topiramate‐ER plus DELI (*N* = 42)	Placebo plus DELI (*N* = 38)	Difference between phentermine‐topiramate‐ER and placebo (95% CI)	Odds ratio	*p*
Primary endpoint					
Body weight change at 3 months—kg	−10.82 (−13.72 to −7.92)	−4.04 (−7.05 to −1.04)	−6.78 (−10.95 to −2.60)		0.002
Key secondary endpoints					
Body weight change at 3 months in completers—kg	−10.61 (−13.54 to −7.67)	−3.84 (−6.94 to −0.74)	−6.77 (−11.04 to −2.50)		0.003
Body weight change at 6 months—kg	−16.19 (−19.28 to −13.11)	−6.40 (−9.69 to −3.10)	−9.80 (−14.31 to −5.28)		< 0.001
Body weight change at 6 months in completers—kg	−17.05 (−20.20 to −13.90)	−6.26 (−9.65 to −2.86)	−10.79 (−15.42 to −6.16)		< 0.001
Body weight change at 9 months—kg	−16.19 (−19.28 to −13.11)	−6.64 (−9.92 to −3.37)	−9.64 (−14.11 to −5.17)		< 0.001
Body weight change at 9 months in completers—kg	−16.16 (−19.26 to −13.06)	−6.31 (−9.67 to −2.94)	−9.85 (−14.42 to −5.28)		< 0.001
Body weight change at 12 months—kg	−15.32 (−18.65 to −12.00)	−5.85 (−9.40 to −2.30)	−9.48 (−14.34 to −4.61)		< 0.001
Body weight change at 12 months in completers—kg	−15.74 (−19.23 to −12.25)	−5.12 (−8.85 to −1.39)	−10.63 (−15.73 to −5.52)		< 0.001
Percent body‐weight loss at 12 months	−15.21 (−16.88 to −13.51)	−4.78 (−6.54 to −3.03)	−10.43 (−12.85 to −8.01)		< 0.001
Percent body‐weight loss at 12 months in completers	−14.30 (−17.09 to −11.51)	−4.49 (−7.48 to −1.51)	−9.81 (−19.03 to −0.59)		< 0.001
Participants with weight reduction of ≥ 5% at 12 months—%	42 (100.0%)	16 (42.1)		115.9 (6.5 to 1000)	< 0.001
Participants with weight reduction of ≥ 10% at 12 months—%	35 (83.3%)	4 (10.5%)		42.5 (11.4 to 158.5)	< 0.001
Participants with weight reduction of ≥ 15% at 12 months—%	18 (42.9%)	2 (5.3%)		13.5 (2.87 to 63.6)	< 0.001
Participants with weight reduction of ≥ 20% at 12 months—%	5 (11.9%)	2 (5.3%)		2.4 (0.44 to 13.4)	0.31
Change from baseline to 12 months					
Waist circumference—cm	−12.6 (−15.8 to −9.41)	−2.07 (−5.48 to 1.35)	−10.53 (−13.8 to −7.23)		< 0.001
Hip circumference—cm	−10.8 (−13.0 to −8.6)	−3.51 (−5.86 to −1.16)	−7.24 (−9.51 to −4.97)		< 0.001
Change in body composition from baseline to 12 months in completers^a^					
Total fat mass—kg	−11.46 (−14.56 to −8.36)	−4.18 (−7.57 to −0.78)	−7.28 (−11.88 to −2.69)		0.004
Total fat mass—%	−5.47 (−7.14 to −3.81)	−2.07 (−3.89 to −0.24)	−3.41 (−5.87 to −0.94)		0.005
Total lean mass—kg	−3.20 (−4.40 to −2.00)	−1.23 (−2.55 to 0.09)	−1.97 (−3.75 to −0.18)		0.023
Total lean mass—%	4.92 (3.08 to 6.75)	1.41 (−0.60 to 3.43)	3.50 (0.78 to 6.23)		0.008
Change in quality of life from baseline to 12 months in completers					
Quality of life (SF12) physical score	1.99 (−0.34 to 4.32)	0.57 (−1.76 to 2.90)	1.42 (−0.23 to 3.07)		0.43
Quality of life (SF12) mental score	0.42 (−1.66 to 2.50)	−1.48 (−3.56 to 0.60)	1.90 (−0.58, 4.39)		0.13

*Note*: All analyses are done by intention to treat, unless indicated otherwise.

Abbreviation: DELI, digitally enhanced lifestyle intervention.

^a^ Body composition was assessed at baseline (randomization visit) and at the 12‐month follow‐up. Measurements were missing at one or both time points for 25 participants (12 in the phentermine–topiramate‐ER group and 13 in the placebo group).

**TABLE 3:** Change in cardiometabolic parameters and wearable‐tracked data at 12 months.^a^

Endpoints	Phentermine‐topiramate‐ER plus DELI (*N* = 42)	Placebo plus DELI (*N* = 38)	Difference between phentermine‐topiramate‐ER and Placebo (95% CI)	*p*
Cardiometabolic parameters				
Systolic blood pressure—mm Hg	−10.36 (−14.72 to −6.01)	−5.98 (−10.65 to −1.31)	−4.39 (−10.77 to 0.76)	0.18
Diastolic blood pressure—mm Hg	−4.79 (−7.22 to −2.35)	−1.16 (−3.78 to 1.45)	−3.62 (−7.20 to −0.05)	0.048
Fasting glucose—mg/Ll	−4.00 (−8.79 to 0.79)	1.77 (−3.40 to 6.95)	−5.77 (−12.82 to 1.28)	0.11
Glycated hemoglobin—%	−0.18 (−0.29 to −0.07)	−0.14 (−0.26 to −0.01)	−0.05 (−0.21 to 0.12)	0.57
Cholesterol—mg/d	9.28 (−31.57 to 50.14)	17.52 (−27.04 to 62.08)	−8.24 (−68.70 to 52.21)	0.79
Triglycerides—mg/dL	−32.65 (−53.83 to −11.47)	−6.39 (−29.45 to 16.66)	−26.25 (−57.56 to −5.05)	0.10
LDL cholesterol—mg/dL	7.38 (0.34 to 14.42)	13.14 (5.47 to 20.81)	−5.76 (−16.17 to 4.65)	0.28
HDL cholesterol—mg/dl	6.02 (1.23 to 10.81)	7.61 (2.37 to 12.84)	−1.59 (−8.68 to 5.50)	0.66
hsCRP—mg/L	−2.61 (−4.46 to −0.77)	−2.27 (−4.29 to −0.25)	−0.34 (−3.07 to 2.39)	0.81
ASCVD 10 years—%^c^	−0.68 (−1.21 to −0.16)	0.00 (−0.23 to 0.23)	−0.68 (−1.12 to −0.24)	0.06
ASCVD lifetime risk—%^c^	−3.35 (−5.98 to −0.72)	3.43 (−0.24 to 7.09)	−6.78 (−9.92 to −3.64)	0.004
Wearable‐tracked data				
Step count—steps/day	1214 (−192 to 2620)	2316 (807 to 3824)	−1102 (−3164 to 960)	0.30
Resting pulse—beats/min	−0.38 (−3.24 to 2.47)	3.05 (−0.03 to 6.13)	−3.43 (−7.63 to 0.76)	0.11
Exercise maximal pulse—beats/min	−4.49 (−9.94 to 0.96)	−3.73 (−9.70 to 2.25)	−0.76 (−8.85 to 7.32)	0.85
Daily calorie expenditure—kcal^b^	−193 (−356 to −30)	60 (−127 to 247)	−252 (−500 to −4)	0.048

^a^ All analyses are done by intention to treat, unless indicated otherwise. DELI denotes digitally enhanced lifestyle intervention, HDL denotes high‐density lipoprotein, LDL denotes low‐density lipoprotein, and hsCRP denotes high sensitivity C‐reactive protein.

^b^ Daily calorie expenditure was estimated using Apple's proprietary heart rate–calorimetry model, which combines personal characteristics (age, sex, weight, and height) with sensor‐derived data (heart rate and accelerometer) collected by the Apple Watch.

^c^ ASCVD scores were calculated using the ASCVD Risk Estimator Plus in a per protocol fashion for patients who had available laboratory data at baseline and at 9 or 12 months. The required parameters for ASCVD risk calculation are available at: https://tools.acc.org/ascvd‐risk‐estimator‐plus/#!/calculate/estimate/. For the 10‐year ASCVD risk score, 23 participants in the phentermine‐topiramate‐ER group and 14 participants in the placebo group had available data. For the lifetime ASCVD risk score, 34 participants in the phentermine‐topiramate‐ER group and 28 participants in the placebo group had available data.

### 3.4 Digitally Enhanced Lifestyle Intervention Parameters

There were no differences in the daily steps, exercise tracked, resting heart rate, number of data points collected with the tracker, and number of recorded weight measurements by the digital scale (Table 3 and Table S4). The phentermine‐topiramate‐ER group had a decrease in the estimated daily calorie expenditure when compared to placebo (difference −252 kcal; 95% CI, −500 to −4; *p* = 0.048). There were no differences in the number of visits (total, in‐person, and telehealth) or the quality of life between both groups.

### 3.5 Side Effects

The phentermine‐topiramate‐ER group had a higher rate of reported adverse events (26%), as compared to the placebo group (8%) (odds ratio: 4.14; 95% CI, 1.06 to 16.21; *p* = 0.04). The most frequently reported side effects were paresthesias, followed by dry mouth and dysgeusia (Table 4). There were no serious adverse events during the study. Two participants (4.8%) in the phentermine‐topiramate‐ER group and one (2.6%) in the placebo group withdrew from the study due to nonserious adverse events and no participant dropped out due to adverse events. Phentermine‐topiramate‐ER was not associated with an increase in resting or exercise heart rate or an increase in systolic or diastolic blood pressure at any point during the study. There were no pregnancies during the study.

**TABLE 4:** Adverse events.

Adverse event	Phentermine‐topiramate‐ER plus DELI (*N* = 42)		Placebo plus DELI (*N* = 38)	
	No. of participants (%)	No. of events	No. of participants (%)	No. of events
Any adverse event	11 (26.2)	17	3 (7.9)	3
Severe adverse events	0 (0)	0	0 (0)	0
Paresthesia	6 (14.3)	6	1 (2.6)	1
Dry mouth	3 (7.1)	3	2 (5.3)	2
Dysgeusia	3 (7.1)	3	0 (0)	0
Insomnia	3 (7.1)	3	0 (0)	0
Headache	2 (4.8)	2	0 (0)	0
Withdrawals related to any adverse event	2 (4.8)	2	1 (2.6)	1
Withdrawals related to serious adverse events	0 (0)	0	0 (0)	0

Abbreviation: DELI, digitally enhanced lifestyle intervention.

## 4 Discussion

In this trial, phentermine‐topiramate‐ER in addition to a DELI program resulted in a mean weight loss of 10.8 kg at 3 months and 15.3 kg at 12 months. This weight loss was 2.6 times greater than the placebo group at both time points. Moreover, 83% of participants in the phentermine‐topiramate‐ER group achieved at least 10% TBWL compared to only 11% of participants in the placebo group. Importantly, phentermine‐topiramate‐ER with a DELI decreased the lifetime ASCVD risk in participants with obesity.

The weight loss observed in this study was substantially greater than the weight loss reported in previous studies with phentermine‐topiramate‐ER [22, 28] and close to the weight loss outcomes of newer AOMs, such as semaglutide [29, 30]. In the pivotal CONQUER trial, participants on the phentermine‐topiramate‐ER 7.5/46 mg dose lost 8.1 kg compared to 1.4 kg in the placebo group at 56 weeks [22]. This substantial difference in weight loss could be attributed to the hybrid visit modality (remote and in‐person) and the incorporation of digital devices to provide actionable feedback based on patients' data, which can lead to better adherence, as suggested by other studies [31, 32].

In previous trials, the combination of AOMs with intense lifestyle interventions resulted in greater weight loss outcomes [33, 34], however, those included approximately 30 visits during their study length, compared to only 16 hybrid visits in this study. Interestingly, in this study, as well as in previous trials of AOMs plus intense lifestyle interventions, participants assigned to placebo also achieved greater weight loss than in trials with less‐intense lifestyle interventions. This difference suggests that the weight loss achieved with AOMs could be enhanced by implementing a DELI program that can be adapted to different health care settings and patient‐specific needs [33, 34, 35].

Although not a novel method of health delivery, hybrid telehealth models have become a widespread practice since the COVID‐19 pandemic. A recent report has highlighted that the use of telehealth for obesity management has the potential to overcome certain barriers to care, including access, stigma, and lack of adherence [10]. Given the widespread adoption of wearables and telehealth and the worsening obesity epidemic, it is crucial to develop cost‐effective programs that enhance weight loss outcomes. Recently, the Institute for Clinical and Economic Review (ICER) report classified phentermine‐topiramate‐ER as the most cost‐effective medication to treat obesity [36]. The use of wearables and digital monitors has shown suboptimal benefits for weight management when added to standard lifestyle interventions [37, 38] or commercial programs [37, 38]. More studies are needed to delineate the key contributors to the synergistic effect observed in this combined therapy and to determine the cost‐effectiveness of a DELI compared to traditional lifestyle intervention with or without AOMs. Interestingly, we observed a sustained > 5% TBWL in the placebo group from month 6 to 12, with a similar dropout rate to that of the phentermine‐topiramate‐ER group. These findings are consistent with other studies comparing digital and traditional interventions [39, 40]. Although the weight loss achieved in the placebo group was less than that seen with newer agents such as semaglutide and tirzepatide, a DELI may represent a viable alternative for individuals with limited access to, or contraindications for, these therapies.

ASCVD is one of the leading preventable causes of death worldwide, with obesity as a key independent risk factor [41]. As demonstrated in the post hoc analyses from the Look AHEAD trial, ≥ 10% TBWL decreases the risk of death from ASCVD in participants with obesity and type 2 diabetes [42]. Here, we report that after 1 year, participants on phentermine‐topiramate‐ER and DELI had a significant decrease of 6% in the lifetime ASCVD risk compared to placebo and DELI. While these findings highlight the importance of weight loss in reducing cardiovascular disease risk, the trajectory of ASCVD risk beyond 12 months and the long‐term impact of weight change on ASCVD risk warrant further investigation.

This study has several strengths. First, the intercalation of virtual and in‐person visits allowed for close and frequent monitoring to help participants reach the lifestyle intervention goals. And second, the use of the digital devices helped participants engage and monitor their own progress. The study was limited by several factors. First, the study was limited by a modest sample size, and while the completion rate at the primary endpoint of 3 months was 90%, the 12‐month results were limited by a 66% completion rate. Importantly, while 26% of participants in the study group reported adverse events, these were minor and similar to prior studies. Second, compliance with the use and data entry of the digital devices was limited by the technology literacy of each patient. Third, while participants were provided with a calorie intake goal, the smartphone application did not include a built‐in calorie tracking option so we were unable to assess individual caloric intake and compliance with the study low‐calorie goals [17]. Finally, our study population was predominantly composed of self‐reported White participants, and the majority were female, which may limit the generalizability of our findings.

## 5 Conclusion

In adults with obesity, adding phentermine‐topiramate‐ER to a DELI led to significantly greater weight loss and a decrease in estimated cardiovascular disease risk compared with DELI and placebo. Further studies are warranted to determine whether a DELI provides an additive or synergistic benefit when combined with pharmacologic weight loss therapies and to evaluate the impact on physical activity, caloric intake, adverse events, and treatment adherence.

## Author Contributions

All authors had full access to all the data and statistical analyses. A.A. had full access to all the data in the study and takes responsibility for the integrity of the data and the accuracy of the data analysis. Concept and design: A.A., M.D.H. Acquisition, analysis, or interpretation of data: A.A., A.C., M.D.H., L.C., W.G., D.S., S.F., D.A., M.L.R.‐S., A.H., M.S. Drafting of the manuscript: A.A., A.C., W.G., M.D.H. Critical revision of the manuscript for important intellectual content: A.A., M.D.H. Statistical analysis: W.S.H., H.J.G. Administrative, technical, or material support: M.S. W.S.H., and H.J.G. are Statisticians and statistical support. C.P., D.L., S.T.V. are Employees from Sponsor (vivus) and supported with study design, interpretation of results.

## Funding

This work was supported by Vivus LLC. The funder did not participate in study design, participant recruitment, collection, analysis, and interpretation of data, writing of the report, or the decision to submit the article for publication.

## Disclosure

Dr. Andres Acosta affirms that this manuscript is an honest, accurate, and transparent account of the study being reported; that no important aspects of the study have been omitted; and that any discrepancies from the study as planned (and, if relevant, registered) have been explained. Data from this trial were also used in a separate analysis, reported in a distinct manuscript, to evaluate weight loss outcomes in relation to a machine‐learning‐assisted genetic risk score for calories‐to‐satiation (CTS_GRS_). This score was developed in an independent participant population and subsequently applied to participants from this trial for external validation [23].

## Conflicts of Interest

A.A. and Mayo Clinic hold equity in Phenomix Sciences Inc. and are inventors of intellectual property licensed to Phenomix Sciences Inc. A.A. served as a consultant for Rhythm Pharmaceuticals, General Mills, Currax, Nestle, Amgen, Structure, and Boehringer Ingelheim. C.P., D.L., and S.T.V. are employees of Vivus LLC. The other authors declare no conflicts of interest.

## Supporting information


**Data S1:** Supporting Information.

## Data Availability

The investigators will share deidentified data that underlie the results reported in this article after deidentification upon request by bona fide researchers who provide a methodologically appropriate proposal. Proposals should be directed to acosta.andres@mayo.edu. To gain access, data requestors will need to sign a data access agreement.
