# Supplementary material for: Addition of Phentermine‐Topiramate to a Digitally Enhanced Lifestyle Intervention: A Double‐Blind Randomized Clinical Trial
Source: Obesity (Silver Spring). 2026 Jan 21;34(3):524–36. doi: 10.1002/oby.70108 (PMC12933223; doi:10.1002/oby.70108)
Supplement: Supplementary file 1 — Data S1: Supporting Information. [file OBY-34-524-s001.zip › Clean R2 SUPPLEMENTARY APPENDIX.docx]

**SUPPLEMENTARY APPENDIX**

CONTENTS

[SUPPLEMENTAL METHODS 2](#_Toc147916583)

[COMPLETE ELIGIBILITY CRITERIA 2](#_Toc147916584)

[Inclusion criteria 2](#_Toc147916585)

[Exclusion criteria 2](#_Toc147916586)

[MEASUREMENTS 3](#_Toc147916587)

[Patient reported outcomes 3](#_Toc147916588)

[Body composition analysis 3](#_Toc147916589)

[STATISTICAL ANALYSIS METHODS 4](#_Toc147916590)

[ENDPOINTS 4](#_Toc147916591)

[Primary endpoint 4](#_Toc147916592)

[Secondary endpoints 4](#_Toc147916593)

[Safety endpoints 5](#_Toc147916594)

[SUPPLEMENTARY TABLES 6](#_Toc147916595)

[Table S1.  Secondary End Points, Cardiovascular and Metabolic changes, and Wearable data (Per Protocol) 6](#_Toc147916596)

[Table S2 Body composition for participants treated with Phentermine – Topiramate ER compared to placebo (Per Protocol) 8](#_Toc147916597)

[SUPPLEMENTARY FIGURES 9](#_Toc147916598)

[Figure S1. Trial design 9](#_Toc147916599)

[Figure S2: CONSORT Flow 10](#_Toc147916600)

[Figure S3: Lifetime Atherosclerotic Cardiovascular Disease risk change with phentermine-topiramate-ER compared to Placebo 11](#_Toc147916601)

[REFERENCES 12](#_Toc147916602)

# SUPPLEMENTAL METHODS

## COMPLETE ELIGIBILITY CRITERIA

### Inclusion criteria

a) Adults with obesity (BMI >30Kg/m2); these will be otherwise healthy individuals with no unstable psychiatric disease and controlled comorbidities or other diseases.

b) Age: 18-75 years.

c) Gender: Men or women. Women of childbearing potential will have negative pregnancy tests within 48 hours of enrollment.

d) Women of childbearing potential must agree to use a method of effective contraception during study participation.

e) Subject must have an Apple iPhone 6s or later with iOS 13 or later and be willing to download the smartphone application (VitalTech Affiliates LLC) application from the Apple App Store.

f) Able to provide written informed consent prior to any study procedures, and be willing and able to comply with study procedures

### Exclusion criteria

a) History of Abdominal bariatric surgery

b) Weight is greater than 450 lbs (204 kg)

c) Recent use (within the last three months) of any antiobesity medication

d) Recent weight change (gain or loss weight greater than 3% TBW in the last 3 months)

e) Positive history of chronic gastrointestinal diseases, or systemic disease that could affect gastrointestinal motility, or use of medications that may alter gastrointestinal motility, appetite or absorption, e.g., orlistat, within the last 6 months.

f) Significant untreated psychiatric dysfunction based upon screening with the Hospital Anxiety and Depression Inventory (HAD), and the Questionnaire on Eating and Weight Patterns (binge eating disorders and bulimia). If such a dysfunction is identified by an anxiety or depression score >11 or difficulties with substance or eating disorders, the participant will be excluded and given a referral letter to his/her primary care doctor for further appraisal and follow-up.

g) Hypersensitivity or contraindication to the study medication.

h) Participant unable or unwilling to follow protocol including use of the wearable activity tracker, digital wellness devices, VitalCare application, or unwilling to sign consent.

i) Principal Investigator discretion

## MEASUREMENTS

### Patient reported outcomes

#### Short Form Survey (SF-12)

The SF-12 is a 12-Iitem tool designed to gather self-reported outcomes assessing the impact of an intervention on daily life, and commonly employed to assess for quality of life. It contains 8 different domains, which are the same as the more extensive form (SF-36):

- Limitations in physical activities because of health problems.
- Limitations in social activities because of physical or emotional problems
- Limitations in usual role activities because of physical health problems
- Bodily pain
- General mental health (psychological distress and well-being)
- Limitations in usual role activities because of emotional problems
- Vitality (energy and fatigue)
- General health perceptions

As well, the SF-12 reproduces the Physical Component Summary and Mental Component Summary scales of the SF-36. The SF-12 has been validated in previous studies, which have showed that results obtained with the SF-12 were consistent with results obtained with the SF-36^1^. As well, it has been shown that the SF-12 is an effective tool for reflecting changes over time^2^.

### Body composition analysis

Body composition was determined at baseline and during the last visit (12 months). We employed the Lunar iDXA (GE Healthcare, Madison, WI, USA) to conduct body composition analysis in our study. All measurements were made at Charlton Clinical Research Unit by a certified research technician .

A full body scan was done, and then analyzed with the encore software (GE Healthcare). The study technician was blinded to group allocation and conducted the studies in an identical fashion for all participants.

Standard regions of interest (ROI) were collected, including android, gynoid, trunk and extremities. The encore software automatically demarcated ROI boundaries, and then this were adjusted by the research technician.

## STATISTICAL ANALYSIS METHODS

### ENDPOINTS

Primary endpoint

- Total body weight loss at 3 months

### Secondary endpoints

- Total body weight loss at 6, 9 and 12 months
- Proportion of participants achieving a total body weight loss percentage of at least 5%, 10% , 15% or 20%>
- Change at 12 months in
  - Vital signs
    - Systolic blood pressure
    - Diastolic blood pressure
  - Body composition analysis assessed by dual energy x-ray absorptiometry (DEXA)
    - Total fat mass and total fat mass percentage
    - Total lean mass and total lean mass percentage
    - Total fat free mass and total fat free mass percentage
  - Anthropometrics
    - Hip circumference
    - Waist circumference
    - Body mass index (BMI)
  - Laboratory parameters
    - Fasting glucose
    - Glycated hemoglobin
    - Triglycerides
    - LDL cholesterol
    - HDL cholesterol
    - High sensitivity C reactive protein
  - Wearable tracked data
    - Daily step count
    - Resting pulse
    - Exercise maximal pulse
    - Calculated daily burned calories
  - ASCVD lifetime and 10-year Risk Score
  - SF-12 scores

### Safety endpoints

- Number of adverse events attributed to the medication from baseline to 12 months
- Number of serious adverse events from baseline to 12 months

## SUPPLEMENTARY TABLES

### Table S1.  Secondary End Points, Cardiovascular and Metabolic changes, and Wearable data (Per Protocol)

| **End Points** | **Phentermine-Topiramate ER Plus DLI  (N=42)** | **Placebo Plus DLI  (N=38)** | **Difference between Phentermine-topiramate ER and Placebo (95% CI)** | **P value** |
| --- | --- | --- | --- | --- |
| **Cardio-metabolic parameters** |  |  |  |  |
| Pulse — beats per min | -0.09 (-3.49 to 3.32) | 3.61 (-0.24 to 7.46) | -3.70 (-8.83 to 1.44) | 0.10 |
| Systolic blood pressure — mm Hg | -13.5 (-18.4 to -8.51) | -10.4 (-15.7 to -5.08) | -3.08 (-10.32 to -4.16) | 0.54 |
| Diastolic blood pressure — mm Hg | -5.48 (-8.32 to -2.64) | -1.89 (-4.93 to 1.15) | -3.59 (-7.76 to -0.57) | 0.15 |
| Fasting Glucose — mg/dl | -1.13 (-5.03 to 2.77) | 1.21 (-3.22 to 5.64) | -2.34 (-8.24 to 3.56) | 0.42 |
| Glycated hemoglobin — % | -0.16 (-0.25 to -0.06) | -0.09 (-0.20 to 0.03) | -0.07 (-52.16 to 10.50) | 0.18 |
| Cholesterol — mg/dl | 2.55 (-28.0 to 33.1) | 41.7 (6.96 to 76.5) | -39.2 (-85.4 to 7.12) | 0.11 |
| Triglycerides — mg/dl | -39.3 (-67.5 to -11.0) | -12.00 (-44.1 to 20.1) | -27.3 (-24.8 to -29.7) | 0.08 |
| LDL cholesterol — mg/dl | 4.62 (-1.97 to 11.2) | 14.8 (7.58 to 22.1) | -10.2 (-20.0 to -0.41) | 0.047 |
| HDL cholesterol — mg/dl | 3.97 (-1.92 to 9.85) | 8.25 (1.56 to 14.9) | -4.28 (-13.2 to -4.62) | 0.66 |
| hsCRP — mg/L | -0.96 (-2.11 to 0.19) | -1.16 (-2.47 to 0.15) | 0.20 (-1.55 to 1.94) | 0.73 |
| **Wearable-tracked data** |  |  |  |  |
| Step count — steps/day | -737 (-1989 to 515) | 280 (-1104 to 1664) | -1017 (-1015 to -1019) | 0.48 |
| Resting pulse — beats per min | -0.09 (-3.49 to 3.32) | 3.61 (-0.24 to 7.46) | -3.70 (-0.93 to -6.46) | 0.10 |
| Exercise maximal pulse — beats per min | -5.26 (-11.2 to 0.68) | -4.18 (-11.1 to 2.74) | -1.08 (-0.63 to -1.54) | 0.40 |
| Calculated daily calories burned — kcal | -200.41 (-432.99 to 32.17) | 200.64 (-90.9 to 492.2) | -401.05 (-397 to -405) | 0.05 |
| HDL denotes high-density lipoprotein, LDL low-density lipoprotein, and hsCRP high sensitivity C-reactive protein. | | | | |

### Table S2 Body composition for participants treated with Phentermine – Topiramate ER compared to placebo (Per Protocol)

Supplementary Table 3: Body composition for participants treated with Phentermine – Topiramate ER compared to placebo

| **DXA Body Composition** | **Phentermine-Topiramate ER Plus DLI** | **Placebo Plus DLI** | **Difference between Phentermine-topiramate ER and Placebo (95% CI)** | **p value** |
| --- | --- | --- | --- | --- |
| Baseline % Android tissue fat^*^ | 56.3±7.1 | 56.7±5.7 |  | 0.79 |
| Difference in % Android Tissue fat at 12 months^¶^ | -7.30 (-9.75 to -4.85) | -2.15 (-4.83 to 0.53) | -5.15 (-8.78 to -1.52) | 0.0042 |
| Baseline % Gynoid Tissue fat^*^ | 50.1±7.1 | 50.1±6.8 |  | 0.97 |
| Difference in % Gynoid Tissue fat at 12 months^¶^ | -4.86 (-6.46 to -3.27) | -1.97 (-3.71 to -0.22) | -2.90 (-5.26 to -0.53) | 0.017 |
| ^*^ For baseline values, mean±standard deviation is presented, 40 participants in the phentermine-topiramate ER group had a body composition analysis, and 35 in the placebo group.  ^¶^ Difference at 12 months is presented for participants that had a baseline and end of study (12 months) body composition analysis, 30 in the phentermine-topiramate ER group and 25 in the placebo group. Data is presented as mean (95% confidence interval). | | | | |

## SUPPLEMENTARY FIGURES

### Figure S1. Trial design


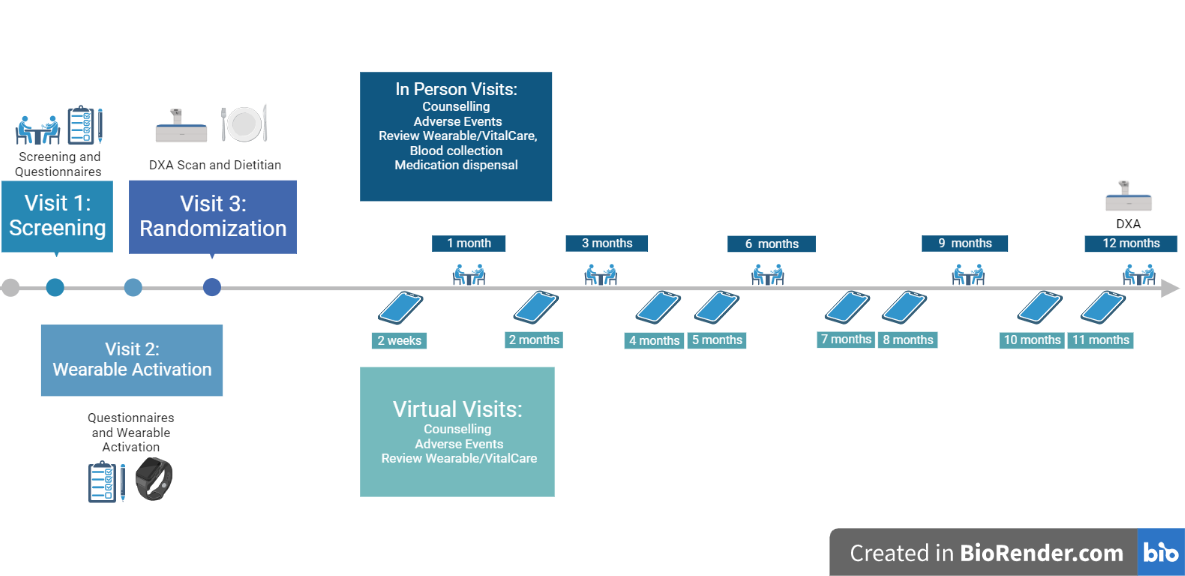


### Figure S2: Lifetime Atherosclerotic Cardiovascular Disease risk change with phentermine-topiramate-ER compared to Placebo


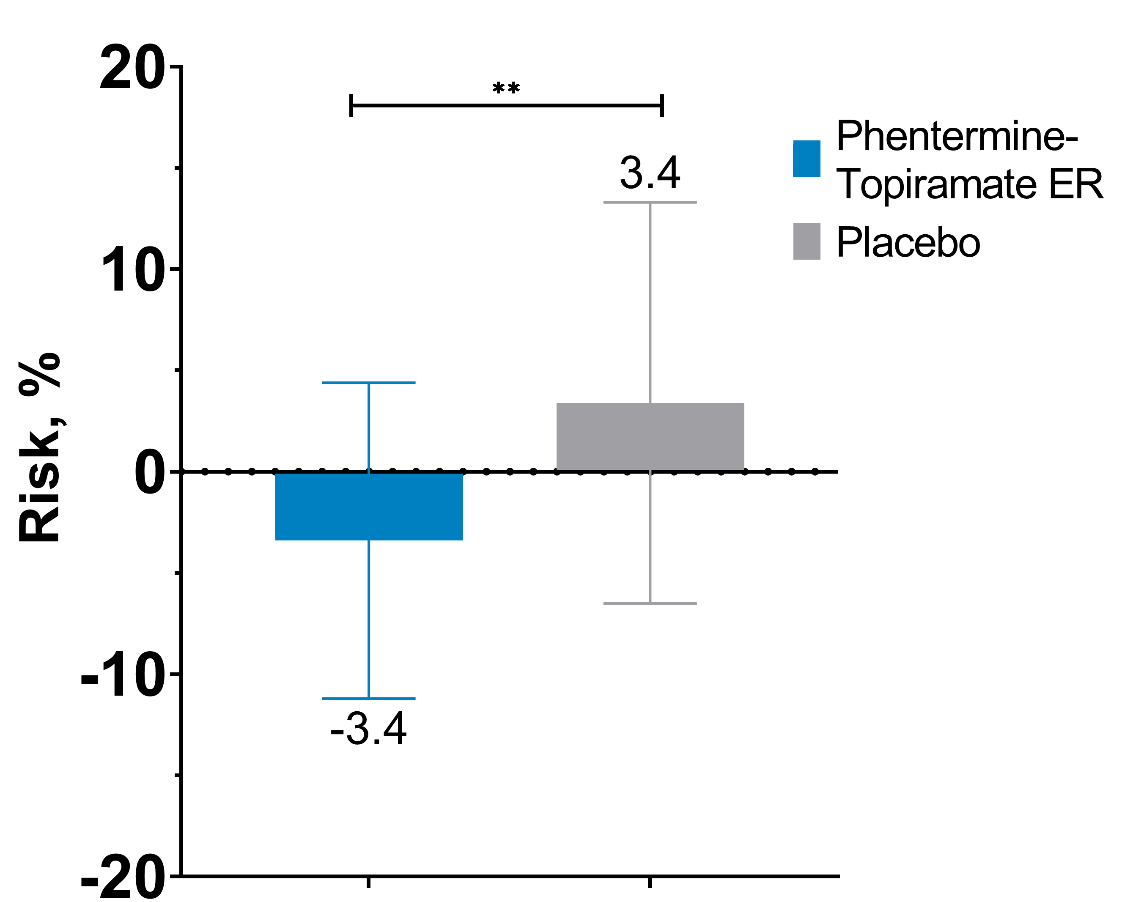


## REFERENCES

1. Ware J, Jr., Kosinski M, Keller SD. A 12-Item Short-Form Health Survey: construction of scales and preliminary tests of reliability and validity. Med Care 1996;34(3):220-33. (In eng). DOI: 10.1097/00005650-199603000-00003.

2. Jenkinson C, Layte R, Jenkinson D, et al. A shorter form health survey: can the SF-12 replicate results from the SF-36 in longitudinal studies? J Public Health Med 1997;19(2):179-86. (In eng). DOI: 10.1093/oxfordjournals.pubmed.a024606.
