# Supplementary material for: Addition of Phentermine‐Topiramate to a Digitally Enhanced Lifestyle Intervention: A Double‐Blind Randomized Clinical Trial
Source: Obesity (Silver Spring). 2026 Jan 21;34(3):524–36. doi: 10.1002/oby.70108 (PMC12933223; doi:10.1002/oby.70108)
Supplement: Supplementary file 1 — Data S1: Supporting Information. [file OBY-34-524-s001.zip › SUPPLEMENTARY MATERIAL PROTOCOL + SAP.docx]

**Supplementary Appendix:**

**Effect of an anti-Obesity Medication Phentermine-Topiramate Extended Release pharmacotherapy vs placebo among patients with obesity using a wearable activity trackers: Randomized, Double-blinded, Placebo-Control, 1-Year, Single-Center Trial**

**Protocol and Statistical Analysis plan**

This supplement contains the following items:

1. Final protocol and statistical analysis plan (version 5; date: 06/22/2021)
2. Protocol amendment history
3. Initial approved protocol and statistical analysis plan (version 1; date: 2/4/2020)
4. Questionnaires and Supplementary Materials

**Effect of an anti-Obesity Medication Phentermine-Topiramate Extended Release pharmacotherapy vs placebo among patients with obesity using a wearable activity trackers: Randomized, Double-blinded, Placebo-Control, 1-Year, Single-Center Trial**

**Principal Investigators:**

- Andres Acosta M.D., Ph.D., Division of Gastroenterology and Hepatology, Department of Medicine, Mayo Clinic, Rochester, MN;

**Co-Investigator:**

- Michael Camilleri, M.D., Division of Gastroenterology and Hepatology, Department of Medicine, Mayo Clinic, Rochester, MN
- Donald Hensrud, M.D., Division of General Internal Medicine, Department of Medicine, Mayo Clinic, Rochester, MN
- Phillip Schulte, Ph.D., Division of Biomedical Statistics & Informatics, Department of Health Sciences Research, Mayo Clinic, Rochester, MN.
- Ryan Lennon, M.S., Division of Biomedical Statistics & Informatics, Department of Health Sciences Research, Mayo Clinic, Rochester, MN.
- Duane Burton, M.S. Division of Gastroenterology and Hepatology, Department of Medicine, Mayo Clinic, Rochester, MN

**Coordinator:**

- Megan Schaefer, Division of Gastroenterology and Hepatology, Department of Medicine, Mayo Clinic, Rochester, MN
- Irene Busciglio, B.S. Division of Gastroenterology and Hepatology, Department of Medicine, Mayo Clinic, Rochester, MN

**Research Fellows:**

- Daniel Gonzalez-Izundegui, M.D., Division of Gastroenterology and Hepatology, Department of Medicine, Mayo Clinic, Rochester, MN
- Sneha Singh M.D. Division of Gastroenterology and Hepatology, Department of Medicine, Mayo Clinic, Rochester, MN
- Angel A. Campos Rodriguez, M.D., Division of Gastroenterology and Hepatology, Department of Medicine, Mayo Clinic, Rochester, MN

**IRB approved:** Pending

**Conflict of Interest:** none

Version 5.0

22 June 2021

# Abstract:

**Introduction:** Obesity prevalence continues to increase worldwide. Estimated costs to the healthcare system are more than $220 billion annually. Obesity severity is associated with higher cardiovascular mortality. FDA-approved medications, devices and surgeries have shown long-term improvements in obesity and diabetes, however, their use (population penetrance) remains low. On the contrary, and despite of contradicting published literature, numerous wearable technologies specific to physical activity and diet are widely adopted with minimal long-term data or benefits. Little is known about the effect anti-obesity pharmacotherapy among patients undergoing lifestyle intervention that includes a consumer-based wearable activity tracker.

**Hypothesis and Aims:** We hypothesize that anti-obesity pharmacotherapy (vs placebo) will have an effect on weight loss among patients using an activity tracker as part of a lifestyle intervention. Thus, we aimed to study in a randomized, double blinded, placebo-control, 1-year, single-center trial the effect of Phentermine-topiramate ER (Anti-obesity Pharmacotherapy) vs placebo among patients with obesity using a wearable activity tracker as part of standard lifestyle intervention.

**Methods/Study Design:** We propose a randomized, single-center trial in 80 patients with obesity to study the Effect of **an** anti-Obesity Medication Phentermine-Topiramate Extended Release vs placebo among patients using a wearable activity tracker in weight loss and obesity related comorbidities in 12 months. All the participants will receive a wearable activity tracker with a Bluetooth scale as part of an intense lifestyle intervention (14 visits total with dietitians, physicians and behavioral therapists (or 6 visits and at least one contact per week virtually with wellness coach) and they will be randomized 1:1 to placebo or Phentermine-topiramate ER (Dosing of 3.75/23 mg daily for 15 days, increased to 7.5/46 mg daily). Participants will be randomized according to a computer generated randomization schedule generated by the study statistician’s office and submitted to the Mayo Clinic CTSA research pharmacy. Allocation will be concealed. Study end-points: a) Primary: Total body weight loss at 3 months among the groups; and b) secondary: Total body weight loss at 6, 9 and 12 months among the groups; number of steps (average per week at 3, 6, 9 and 12 months among the groups); calories tracked: number times recorded, calories per day (average per week at 3, 6, 9 and 12 months among the groups), number of exercise sessions (average per week at 3, 6, 9 and 12 months among the groups); hours/week using app/tracker (average per week at 3, 6, 9 and 12 months among the groups); weight loss difference in clinic vs. Bluetooth scale (weight loss difference 3, 6, 9 and 12 months); Quality of life SF36 (at 3, 6, 9 and 12 months among the groups); improvement in obesity-related comorbidities (diabetes/HbA1c, hypertension/SBP-DBP, hyperlipidemia/TC-LDL-HDL-Tg, Sleep apnea/CPAP, Joint Disease/pain).

**Protocol**

**Effect of a Consumer-Based Wearable Activity Tracker in Combination with an Anti-Obesity Pharmacotherapy in Obesity: Randomized, Placebo-Control, 1-Year, Single-Center Trial**

Table of Contents

[Abstract: 2](#_Toc26799503)

[Introduction: 4](#_Toc26799504)

[Treatment for obesity: 4](#_Toc26799505)

[Pharmacotherapy 5](#_Toc26799506)

[Phentermine-Topiramate Extended Release: 5](#_Toc26799507)

[Wearable Activity Trackers 6](#_Toc26799508)

[Hypothesis and Aims: 8](#_Toc26799509)

[Methods/Study Design: 8](#_Toc26799510)

[Randomization and Allocation 12](#_Toc26799511)

[Selection Participants 12](#_Toc26799512)

[Anthropometrics and Metabolic Characteristics studies 13](#_Toc26799513)

[Intense Lifestyle Intervention and Behavioral Treatment 14](#_Toc26799514)

[Statistical Considerations 15](#_Toc26799515)

[Statistical Analysis: 16](#_Toc26799516)

[Anticipated results and significance: 17](#_Toc26799517)

[Potential pitfalls, precautions taken, and alternative strategies: 17](#_Toc26799518)

[References 19](#_Toc26799519)

# Introduction:

Obesity prevalence continues to increase worldwide[2] and, in the United States, 69% of adults are overweight or with obesity[3]. Estimated costs to the healthcare system are more than $480 billion annually. Increased severity of obesity correlates with a higher prevalence of the associated co-morbidities. Likewise, obesity increases the risk of premature mortality [4]. Obesity affects almost every organ system in the body and increases the risk of numerous diseases including type 2 diabetes mellitus, hypertension, dyslipidemia, cardiovascular disease, and cancer. It is estimated that a man in his twenties with a BMI over 45 will have a 22% reduction (13 years) in life expectancy.

***Obesity*** is defined as the amount of excess of adipose tissue at which health risks increase[5]. Normal weight, overweight and obesity can be measured by Body Mass Index (BMI). BMI is calculated by weight (kg) divided by the square of the height (m^2^). The BMI for an adult healthy weight is from 18.5 to 24.9 kg/m^2^, overweight is from 25 to 29.9 kg/m^2^ and obese is 30 kg/m^2^ or above. Obesity is considered morbid or severe when BMI is higher than 40 kg/m^2^ (Guidelines 1998). In children, obesity is measured as BMI higher than the 95^th^ percentile related to their age and sex [6, 7]. Obesity can also be measured by waist circumference defined as larger than 102 cm in men and 88 cm in women. BMI and waist circumference are associated with health risks and obesity–related co-morbidities.

**The Obesity Epidemic**: Obesity has reached epidemic proportions in developed countries and its prevalence is increasing in developing countries [7]. In the United States of America (USA), the prevalence of overweight adults is 64%, and obese adults is 30.5% [8]. In children and adolescents, the obesity prevalence has increased to 17.1% [6]. The World Health Organization indicated that globally, in the year 2005, there were approximately 1.6 billion overweight adults, and of those, 400 million were obese [9]^.^ This alarming obesity epidemic poses a heavy burden to the U.S. economy, costing more than $150 billion every year—10 percent of the total health budget—according to the Centers for Disease Control and Prevention (CDC 2012). In 2009, the U.S. Agency for Healthcare Research and Quality reported that overweight and obese people spend 43 percent more each year in medical expenses than those who have normal weight. The Agency also found that obese workers are paid less than their coworkers who have normal weight [10].

## Treatment for obesity:

The 2013 Obesity Guidelines suggest that to achieve weight loss, an energy deficit is essential. Reducing dietary energy intake below that required for energy balance can be achieved through a reduction of daily calories to 1200-1500 for women, and 1,500-1800 for men (kilocalorie levels are usually adjusted for the individual’s body weight and physical activity levels); or estimation of individual daily energy requirements and prescription of an energy deficit of 500 kcal/d or 750 kcal/d. Recommendations for young children through adolescence vary in order to support normal growth and development occurring during these years. The Academy of Nutrition and Dietetics Evidence Analysis Library recommends no fewer than 900 kcal/day for 6-12 year olds who are medically monitored and no fewer than 1200 kcal/day for 13-18 year olds (Academy of Nutrition and Dietetics Weight Management Position Paper which provides an overview of a nutrition assessment: http://www.eatrightpro.org/resource/practice/position-and-practice-papers/position-papers/weight-management). Evidence supports greatest long-term success with an individualized, structured meal plan in place. A registered dietitian nutritionist can play an important role in designing the nutrition intervention tailored to address each patient’s unique needs and circumstances, taking into consideration factors such as insulin resistance. Any diet program that meets this required energy deficit is appropriate to adopt, and comparative trials have shown no long-term superiority between different macronutrient composition or elimination diets. Furthermore, it is important to adhere to a balanced diet that provides a variety of items from all food groups and limits potentially harmful food ingredients like added sugars, sodium and alcohol. Additionally, guidelines recommend limiting or avoiding liquid calories (i.e. sodas, juices, alcohol, etc.). And, finally, the meal plan should be designed in such a way that the individual is likely to follow it.

Along with the prescription for a reduced calorie diet, a comprehensive lifestyle intervention program should prescribe increased aerobic physical activity (such as brisk walking) for ≥150 min/week (equal to ≥30 min/d most days of the week), and a goal of >10,000 steps per day. Higher levels of physical activity, approximately 200 to 300 min/wk., are recommended to maintain the weight lost or minimize weight regain in the long term (>1 year) [11]. The diet and physical activity can be in combination with a hospital/university or commercial behavior program; these are comprehensive lifestyle interventions that usually provide structured behavior strategies to facilitate adherence to diet and activity recommendations. These strategies include regular self-monitoring of food intake, body weight, physical activity, and food cravings. These same behaviors are recommended to maintain lost weight, with the addition of frequent (i.e., weekly or more frequent) monitoring of body weight[12].

## Pharmacotherapy

In addition to diet, exercise and behavioral modification, pharmacotherapies should be considered as an adjunct to lifestyle changes in patients who have been unable to lose and maintain weight with diet and exercise alone. They should also be considered in people whose history or clinical circumstances require expedited weight loss. Medication should not be used alone, but in combination with an intensive lifestyle program.

Pharmacotherapy for the treatment of obesity can be considered if a patient has a body mass index (BMI) ≥ 30 kg/m^2^ or a BMI ≥ 27 kg/m^2^ with weight-related co-morbidities such as hypertension, type 2 diabetes, dyslipidemia and obstructive sleep apnea[12]. Medical therapy should be initiated with dose escalation based on efficacy and tolerability to the recommended dose. An assessment of efficacy and safety at least monthly for the first three months and then at least every three months. In patients who have cardiovascular disease, guidelines recommend against prescribing sympathomimetic agents such as phentermine and phentermine/topiramate extended release (ER). Lorcaserin and orlistat are safer alternatives. In patients with T2DM, the guidelines suggest antidiabetic agents that promote weight loss such as glucagon-like peptide (GLP-1) analogs which reduce hyperglycemia in addition to the first-line agent for T2DM, metformin[13].

| **Medication / dose** | **Clinical data** | **Mean weight change from baseline after 1 year** | **Weight loss after 1 year (Proportion of participants)** | | | **References** |
| --- | --- | --- | --- | --- | --- | --- |
|  |  |  | **>5%** | **>10%** | **>15%** |  |
| Orlistat  120 mg TID | Clinical data from three trials | −6.0 to 10.3  Kg vs −2.6 to 6.1 Kg with placebo | **36–67%**  (vs.16–43.6%) | **17 - 38.9**  (vs. 8.8 – 24.8) | **NA** | [14-16] |
| Phentermine/ topiramate ER 15 mg/92 mg QD | 1‐year trial, people with obesity (BMI ≥35 kg/m^2^) | −10.9% vs −1.6% with placebo | **70%**  (vs.21%) | **48%**  (vs. 7%) | NA | [17] |
| Lorcaserin  10 mg BID | 2‐year trial, people with obesity or overweight and ≥1 comorbidity | −5.8% vs −2.5% with placebo | **47%**  (vs. 23%) | **22.6**  (vs. 7.7) | NA | [18] |
| Naltrexone/ bupropion SR  32 mg/360 mg | Four 56‐week trials, people with obesity and ≥1 comorbidity | −5.4% vs −1.3% with placebo (COR‐I) | **42%**  (vs. 17%) | **28.3**  (vs. 5.7) | **13.5**  (vs. 2.4) | [19] |
| Liraglutide  3.0 mg QD | 56‐week trial, people with obesity or overweight and ≥1 comorbidity | −7.4% vs −3.0% with placebo | **62%**  (vs. 34%) | **33.1%** (vs. 10.6%) | **14.4%**  (vs. 3.5%) | [20] |

Phentermine-Topiramate Extended Release: When low-dose, controlled-release, phentermine was combined with the glutamatergic and GABA-ergic antiepileptic topiramate in a large phase III study (more than 1400 participants on treatment arms with different doses), subjects lost 10.2 kg on 15/92 mg combination therapy vs. 1.4 kg on placebo over 56 weeks [17]. The most common adverse events were dry mouth, paresthesias, constipation, insomnia, dizziness, and dysgeusia. Depression- and anxiety-related adverse events were also observed. The medication had favorable effects on glycemia, including prevent progression to diabetes, improvements in lipids, blood pressure, sleep apnea, and quality of life measures. There was also, as previously noted, a small but consistent increase in pulse rate [21]. The overall rate of adverse effects decreased in weeks 56–108 compared to weeks 0–56; among which dry mouth, constipation and paresthesias were the most prevalent There were 19 pregnancies carried to term during these studies none of which resulted in congenital abnormalities [21-23].


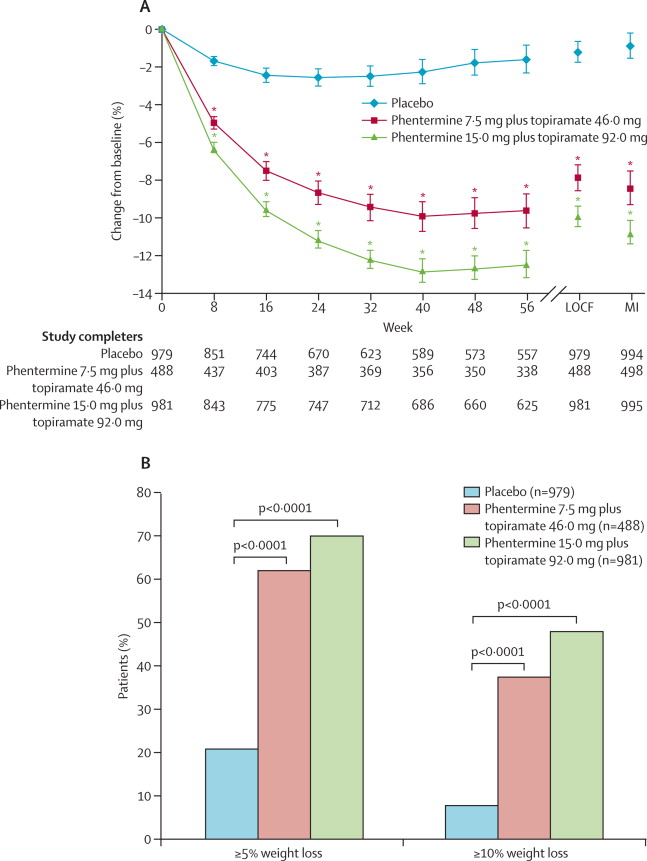


Figure 1. Effects of phentermine plus topiramate on bodyweight. Patients with at least 5% and at least 10% weight loss. [1]

In July 2012, the FDA voted for approval of phentermine (3.75–15mg/d) plus extended release topiramate (23–92mg/d) as an adjunct to diet and physical activity for treatment of obesity among adult individuals with BMI≥30kg/m^2^ or BMI≥27kg/m^2^ with at least one obesity-related comorbid condition. The drug will carry a warning of potential increased risk for orofacial clefts in neonates exposed to topiramate during the first trimester of gestation and will be subject to a Risk Evaluation and Mitigation Strategy (REMS) that will restrict prescribing to trained clinicians, will require effective contraception and monthly pregnancy tests for reproductive age women, and will restrict dispensing to specific mail-order pharmacies. The company is also required to carry a long-term cardiovascular outcomes trial. No randomized pediatric studies have as yet been reported. Noteworthy, the high dose of PhenTop was associated with a mean weight loss of 9.8%; however, only 48% of patients lost >10% of their body weight, and 30% of patients lost <5% of their body weight (Figure 1).

## Wearable Activity Trackers

Consumer-based wearable activity trackers are now readily available and can provide individuals with the ability to objectively monitor their physical activity levels. In addition, when combined with the use of smartphone and computer apps, they may assist users through a range of motivational and tracking tools to better manage their personal health[24]. In addition to providing real-time feedback relating to daily steps and energy expenditure, consumer-based wearable activity trackers have the potential to provide specific, tailored feedback through specifically designed algorithms or by health professionals. This type of emerging technology may provide an alternative means of providing ongoing support and motivation to individuals both looking to increase their activity levels or to maintain activity levels following a structured lifestyle intervention[25]. Moreover, consumer-based wearable activity trackers may assist in reducing the resource and time burden associated with traditional methods of providing ongoing support. Randomized controlled trials have shown that these devices have promise in relation to increasing physical activity participation[26, 27]; however, participant numbers in individual studies tend to be low, making it difficult to adequately assess the benefits of these devices. Furthermore, there is limited research relating to their long-term adherence and effectiveness.

A recent systematic review[28] of that aimed to determine the effects of interventions utilizing consumer-based wearable activity trackers on physical activity participation and sedentary behavior when compared with interventions that do not utilize activity tracker feedback, showed that there was a significant increase in daily step count (standardized mean difference [SMD] 0.24; 95% CI 0.16 to 0.33; P<.001), moderate and vigorous physical activity (SMD 0.27; 95% CI 0.15 to 0.39; P<.001), and energy expenditure (SMD 0.28; 95% CI 0.03 to 0.54; P=.03) and a nonsignificant decrease in sedentary behavior (SMD −0.20; 95% CI −0.43 to 0.03; P=.08) following the intervention versus control comparator across all studies in the meta-analyses. In general, included studies were at low risk of bias, except for performance bias. Heterogeneity varied across the included meta-analyses ranging from low (I2=3%) for daily step count through to high (I2=67%) for sedentary behavior. Utilizing a consumer-based wearable activity tracker as either the primary component of an intervention or as part of a broader physical activity intervention has the potential to increase physical activity participation. As the effects of physical activity interventions are often short term, the inclusion of a consumer-based wearable activity tracker may provide an effective tool to assist health professionals to provide ongoing monitoring and support[28].

Online support system: VitalCare (VitalTech Affiliates LLC) is a digital health platform that allows remote collection of date from the wearable tracker and digital wellness devices used as part of this study. It also will allow subjects to document study medication compliance and will allow the remote visits to be conducted through a video conference between subjects and appropriate study team members.

FDA-approved medications, devices and surgeries have shown long-term improvements in obesity and diabetes, however, their use (population penetrance) remains low (less than 1% market use). On the contrary, and despite of contradicting published literature, numerous wearable technologies specific to physical activity and diet are widely adopted with minimal long-term data or benefits. Little is known about the effect anti-obesity pharmacotherapy among patients undergoing lifestyle intervention that includes a consumer-based wearable activity tracker.

# Hypothesis and Aims:

We hypothesize that anti-obesity pharmacotherapy (vs placebo) will have an effect on weight loss among patients using an activity tracker as part of a lifestyle intervention. Thus, we aimed to study in a randomized, double-blinded, placebo-control, 1-year, single-center trial the effect of Phentermine-topiramate ER (Anti-obesity Pharmacotherapy) vs placebo among patients with obesity using a wearable activity tracker as part of standard lifestyle intervention.

# Methods/Study Design:

We propose a randomized, double blinded, single-center trial in 80 patients with obesity to study the Effect of an anti-Obesity Medication Phentermine-Topiramate Extended Release vs placebo among patients using a wearable activity trackers in weight loss and obesity related comorbidities in 12 months. All the participants will receive a wearable activity tracker and digital wellness devices (Bluetooth scale, Bluetooth pulse oximeter and Bluetooth blood pressure monitor) as part of an intense lifestyle intervention (8 in person visits total with dietitians, physicians and other study team members. At least one contact per month virtually with a member of the study team when an in person visit is not scheduled. Subjects will be randomized 1:1 to placebo or Phentermine-topiramate ER (Dosing of 3.75/23 mg daily for 15 days, increased to 7.5/46 mg daily). Participants will be randomized according to a computer generated randomization schedule generated by the study statistician’s office and submitted to the Mayo Clinic CTSA research pharmacy. Allocation will be concealed. Study end-points: a) Primary: Total body weight loss at 3 months among the groups; and b) secondary: Total body weight loss at 6, 9 and 12 months among the groups; number of steps (average per week at 3, 6, 9 and 12 months among the groups); calories tracked: number times recorded, calories per day (average per week at 3, 6, 9 and 12 months among the groups), number of exercise sessions (average per week at 3, 6, 9 and 12 months among the groups); hours/week using app/tracker (average per week at 3, 6, 9 and 12 months among the groups); weight loss difference in clinic vs. Bluetooth scale (weight loss difference 3, 6, 9 and 12 months); Quality of life SF36 (at 3, 6, 9 and 12 months among the groups); improvement in obesity-related comorbidities (diabetes/HbA1c, hypertension/SBP-DBP, hyperlipidemia/TC-LDL-HDL-Tg, Sleep apnea/CPAP, Joint Disease/pain).

Study flowsheet:

Recruitment 100 subjects

Randomization 80 subjects

Digital support + Placebo n = 40

Digital Support + Phentermine-Topiramate ER n = 40

Visit schedule:

1. Visit 1-Screening visit
2. Visit 2-Baseline anthropometric and metabolic studies
3. Visit 3-Counseling / Randomization / wearable tracker, digital wellness devices and Medication assignment and disbursement.
4. Remote visit-2-week follow up (Remote visit)
5. Visit 4-4-week follow up
6. Remote visit-2 month
7. Visit 5-3 month follow up / samples collection / body composition
8. Remote visit-4 month
9. Remote visit- 5 month
10. Visit 6- 6 month follow up
11. Remote visit-7 month
12. Remote visit-8 month
13. Visit 7-9 month follow up
14. Remote visit-10 month
15. Remote visit-11 month
16. Visit 8-12 month End of study

|  |  |  |  | Months | | | | | | | | | | | | |
| --- | --- | --- | --- | --- | --- | --- | --- | --- | --- | --- | --- | --- | --- | --- | --- | --- |
|  |  |  |  | 1 | | 2 | 3 | 4 | 5 | 6 | 7 | 8 | 9 | 10 | 11 | 12 |
| Study Procedures | Visit 1 Screening | Visit 2 Baseline Studies | Visit 3  Counseling and Randomization | Remote Study Visit  2-Week  (+/- 3 days) | Visit 4  4-Week  (+/- 3 days) | Remote Study Visits | Visit 5 | Remote Study Visits | Remote Study Visits | Visit 6 | Remote Study Visits | Remote Study Visits | Visit 7 | Remote Study Visits | Remote Study Visits | Visit 8 |
| Informed Consent | X |  |  |  |  |  |  |  |  |  |  |  |  |  |  |  |
| Medical History and Physical Examination | X |  |  |  |  |  |  |  |  |  |  |  |  |  |  |  |
| Pregnancy Test | X* | X* | X* |  | X* |  | X* |  |  | X* |  |  | X* |  |  | X* |
| Vital Signs | X | X | X | X | X |  | X |  |  | X |  |  | X |  |  | X |
| Metabolic Studies |  | X |  |  |  |  |  |  |  |  |  |  |  |  |  | X**** |
| Blood Collection |  | X |  |  |  |  | X** |  |  | X** |  |  | X** |  |  | X** |
| Medication Diary |  |  |  |  |  |  |  |  |  |  |  |  |  |  |  |  |
| Dispense medication |  |  | X |  |  |  | x |  |  | x |  |  | x |  |  |  |
| Medication Reconciliation |  |  | X |  |  |  | X |  |  | X |  |  | X |  |  |  |
| Wearable Tracker and Digital Wellness Devices Given |  |  | X |  |  |  |  |  |  |  |  |  |  |  |  |  |
| Randomization and Medication/Placebo Prescription |  |  | X |  |  |  |  |  |  |  |  |  |  |  |  |  |
| Behavioral Questionnaires | X*** | X^a^ |  |  |  |  |  |  |  |  |  |  |  |  |  | X |
| Stool Sample |  | X |  |  |  |  |  |  |  |  |  |  |  |  |  |  |
| Adverse Event Assessment |  |  |  | X | X | X | X | X | X | X | X | X | X | X | X | X |
| Review VitalCare Measurments/pregnancy test results |  |  |  | X | X | X | X | X | X | X | X | X | X | X | X | X |
| Lifestyle Intervention Review |  |  | X |  |  |  |  |  |  |  |  |  |  |  |  |  |
| Home Urine Pregnancy Tests Given |  |  |  |  | X |  | X |  |  | X |  |  | X |  |  |  |

* may be done up to 48 hours prior to visit.

**fasting blood draw only (basic metabolic panel, lipid panel, HbA1C, hsCRP, plasma hormones and proteomics.

***HADS, AUDIT-C and Eating disorders questionnaire only

**** Body composition, fasting blood for basic metabolic panel, lipid panel, HbA1C, hsCRP, metabolomics, plasma hormones and proteinomics

^a^ All questionnaires except HADS, AUDIT-C and Eating disorders questionnaire

^b^ Visit window for visits 5, 6, 7, 8 and all remote visit is +/- 5 days.

## Randomization and Allocation

A computer generated randomization schedule generated by the study statistician’s office will be submitted to the Mayo Clinic CCaTS Research Pharmacy. Randomization will be based on guiding pharmacotherapy or placebo. Allocations will be concealed. This study will be blinded until data are transmitted to the statistician for data lock. All subjects will be given a verbal explanation of the study, provided time to read and study the written consent form and its information, given opportunities to ask questions and a copy of the consent form. Participants will be informed of their right to withdraw from the study at any time without prejudice to their clinical management now or in the future. Consent will be sought by one of the medical doctor investigators or the study coordinator, and consent will be documented by the participant’s signature on the consent form. Mayo’s Institutional Review Board will approve the process and protocol. All the members of multidisciplinary team for weight management (i.e. physicians, coordinators, clinical assistants, registered dietitians will remain blinded).

If unblinding is needed for subject safety the Principal Investigator or a Co-Investigator will contact a research Pharmacist or the research pharmacy manager and provide documentation of reason for study unblinding and the subject will be withdrawn from the study.  The study team will document and report the reason for unblinding as required by the Mayo Clinic IRB.

## Selection Participants

We plan to study a cohort of 80 patients with obesity (BMI>30 kg/m^2^). Participants will be recruited from the Mayo Clinic Weight Management and Nutrition Clinic, media advertising, classified ads, and existing databases of patients with obesity, including the phenome registry (Mayo Clinic IRB number 19-000030), the Mayo Clinic biobank and the right-10k cohort.

Inclusion criteria

1. Adults with obesity (BMI >30Kg/m^2^); these will be otherwise healthy individuals with no unstable psychiatric disease and controlled comorbidities or other diseases.
2. Age: 18-75 years.
3. Gender: Men or women. Women of childbearing potential will have negative pregnancy tests within 48 hours of enrollment.
4. Women of childbearing potential must agree to use a method of effective contraception during study participation.
5. Subject must have an Apple iPhone 6s or later with iOS 13 or later and be willing to download the VitalCare (VitalTech Affiliates LLC) application from the Apple App Store.
6. Able to provide written informed consent prior to any study procedures, and be willing and able to comply with study procedures

Exclusion criteria

1. History of Abdominal bariatric surgery
2. Weight is greater than 450 lbs (204 kg)
3. Recent use (within the last three months) of any antiobesity medication
4. Recent weight change (gain or loss weight greater than 3% TBW in the last 3 months)
5. Positive history of chronic gastrointestinal diseases, or systemic disease that could affect gastrointestinal motility, or use of medications that may alter gastrointestinal motility, appetite or absorption, e.g., orlistat, within the last 6 months.
6. Significant untreated psychiatric dysfunction based upon screening with the Hospital Anxiety and Depression Inventory (HAD), and the Questionnaire on Eating and Weight Patterns (binge eating disorders and bulimia). If such a dysfunction is identified by an anxiety or depression score >11 or difficulties with substance or eating disorders, the participant will be excluded and given a referral letter to his/her primary care doctor for further appraisal and follow-up.
7. Hypersensitivity or contraindication to the study medication.
8. Participant unable or unwilling to follow protocol including use of the wearable activity tracker, digital wellness devices, VitalCare application, or unwilling to sign consent.
9. Principal Investigator discretion

## Anthropometrics and Metabolic Characteristics studies

Anthropometrics Measurements: will be taken of height, weight, blood pressure, pulse, waist and hip ratio, respiration rate and temperature at screening, baseline, randomization day and visit 5, 6, 7, 8 and 9.

Baseline Characteristic studies:

All participants will complete the baseline assessment at the Mayo Clinic after an 8‑hour fasting period, and the following characteristics will be measured at baseline: Fasting blood collection, body composition, resting energy expenditure, gastric emptying with meal for breakfast, behavioral questionnaires, exercise capacity and performance and buffet meal test for lunch. Blood will be collected for assessment of metabolomic biomarkers, gastrointestinal hormones, DNA (blood). Stool samples for microbiome and bile acid. Participants will return to the CRTU to pick up medication based on the randomization, the wearable activity tracker and the digital wellness devices.

If participants have already completed the baseline assessment at the Mayo Clinic within the last 3 years, those participants will not need to complete the full baseline assessment. Participants will complete the baseline assessment at the Mayo Clinic after an 8-hour fasting period again, but only the following characteristics will be measured at baseline: Fasting blood collection and body composition. Blood will be collected for assessment of metabolomics biomarkers, gastrointestinal hormones, DNA (blood). Stool samples for microbiome and bile acid. Participants will return to the CRTU to pick up medication based on the randomization, the wearable activity tracker, and the digital wellness devices.

Methods of metabolic studies

1. Body composition will be measured by DEXA (dual energy x-ray absorptiometry).
2. Resting energy expenditure was assessed by indirect calorimetry with a ventilated hood (Parvo Medics, Sandy, UT).
3. Gastric emptying (GE) of solids by scintigraphy: The primary endpoint is gastric half-emptying time (GE t_1/2_) [29-31]. Images will be acquired at 0, 60, 120 and 240 minutes following the normal clinical gastric emptying testing protocol without a push meal.
4. Appetite (hunger level) by visual analog score fasting and after standard meal for GE and prior to the Satiation test [30].
5. Satiation will be measure by *ad-libitum* buffet meal to measure total caloric intake and macronutrient distribution in the chosen food. Satiation will be reported in calories consumed at fullness (satiation) [30].
6. Satiety by visual analog score postprandial after standard meal for GE and after to the *ad*-libitum buffet meal test for every 30 minutes for 2 hours [30]. Satiety will be measured in length of time of fullness.
7. Samples collection, handling and storage: Samples will be collected after an overnight fast (of at least 8 hours) in the morning. Plasma will be preserved following standard guidelines and protein degradation inhibitors, kalikrein and DPP-IV inhibitors will be added to preserve the samples. Samples will be stored at -80ºC in the PI’s laboratory. Fasting samples will be collected and measure on subsequent visits (every 3 months).
   1. Fasting Blood will be collected for basic metabolic panel, lipid panel, HbA1C, hsCRP
   2. Plasma hormones and proteomics by radioimmunoassay and/or mass spectrometry measured fasting, and postprandial 15, 45, and 90 minutes, with the primary endpoint being the peak postprandial level (test should be done simultaneously to GE). At the end of study visit (Visit 8), this will be drawn once and not at intervals.
   3. Targeted Metabolomics: We will perform quantitative, targeted metabolomics of salient classes of compounds in plasma samples using mass spectrometry.
   4. Blood DNA for genome wide association studies (GWAS)
   5. Stool will be collected and stored to study microbiome, short chain fatty acids and bile acids.
8. Self-administered questionnaires assessing affect, physical activity levels, attitudes, body image, diet, and eating behavior; details of each questionnaire are provided below. Participants will complete a series of questionnaires.
9. Hospital Anxiety and Depression Scale: HADS will be used to screen for severe anxiety or depression.
10. AUDIT-C Alcoholism Screening Test [32] - The AUDIT-C is a 3-item alcohol screening questionnaire that reliably identifies participants who are hazardous alcohol drinkers or have active alcohol use disorders. This score will be used in screening by the study physician/nurse coordinator. The AUDIT-C is scored on a scale of 0-12. Each AUDIT-C question has 5 answer choices. Points allotted are: a=0 points; b=1 point; c=2 points; d=3 points; e=4 points. In men, a score of 4 or more is considered positive, optimal for identifying hazardous drinking or active alcohol use disorders. In women, a score of 3 or more is considered positive (same as above).
11. Eating Disorders Questionnaire - The Questionnaire on Eating and Weight Patterns-Revised [33], is a valid measure of screening for eating disorders which has been used in several national multi-site field trials. Respondents are classified as binge eating disorder, purging bulimia nervosa, non-purging bulimia nervosa, or anorexia nervosa. We have used this instrument to screen for eating disorders in obese populations.
12. Three Factor eating questionnaire is 21-item questionnaire, validated, to assess for emotional eating disorders and food cravings.
13. Physical Activity Level - The four-item Physical Activity Stages of Change Questionnaire [34]will be utilized to assess the physical activity level of participants. Mayo Clinic investigators, led by co-investigator Dr. Clark, have used these items to explore the relationship between quality of life and physical activity in an NCI-funded study on long-term lung cancer survivors [34].
14. Exercise behavior*-* The Exercise Regulations Questionnaire (BREQ-3)*[35]* and its subsequent modifications have become the most widely used measures of the continuum of behavioural regulation in exercise psychology research. It has been used either as a multidimensional instrument giving separate scores for each subscale, or as a unidimensional index of the *degree* of self-determination.
15. SF-12v2- a common questionnaire used to measure quality of life
16. STOP-BANG- questionnaire used to screen for obstructive sleep apnea
17. WOMAC- questionnaire used to assess osteoarthritis of the knee and hip

### Intense Lifestyle Intervention and Behavioral Treatment

All the participants will meet the multidisciplinary team which consists of an Obesity Expert physician, registered dietitian nutritionist as standard of care in our clinical practice. All participants will guided to 1) Nutrition: Reduce dietary intake below that required for energy balance by consuming 1200calories per day for women and 1400calories per day for men; 2) Physical Activity: reach the goal of 10,000 steps or more per day; 3) Exercise: reach the goal of 150 minutes or more of cardiovascular exercise/week; 4) Limit consumption of liquid calories (i.e. sodas, juices, alcohol, etc.). All participants will receive a personal fitness tracker, where their activity and calories will be tracked. This information will be given in a booklet format.

**Wearable tracker**:

Subjects in the study will be provided with an Apple watch Series 5. The Apple watch will be connected by Bluetooth technology to the subject’s personal Apple iPhone. Subjects will be required to use the watch during the study.

**Digital Wellness Devices**:

Subjects in the study will be provided and a wireless scale, automated blood pressure cuff and pulse oximeter. These digital wellness devices will be connected by Bluetooth technology to the subject’s personal Apple iPhone.

Subjects will be allowed to keep the wearable activity tracker and the digital wellness devices if they complete the study. Subjects who withdraw from the study or are withdrawn from the study will be asked to return the digital wellness devices. Subjects will also be provided with a backpack to transport the wellness devices.

**Virtual Care:**

As part of the study subjects will be asked to download the VitalCare (VitalTech Affiliates LLC) application to the subject’s personal Apple iPhone from the Apple App Store. This application will allow the study team to conduct the remote study visits. The application will also be used to monitor subject compliance with taking the study medication and to send reminders to the subject to take the study medication. Additionally this application will record the measurements collected by the digital wellness devices given to the subjects as part of the study. The study subjects will not be required to use the digital wellness devices at any set time points, but it will be encouraged. The digital wellness devices will be used at the subject’s discretion. Any measurements recorded by the digital wellness devices will only be reviewed by the study team during the subject’s study visits, either in person visits or remote visits. Study team will not monitor the use or results of this study until the next schedule visit or until the participant brings it to our attention.

**Medication**

Medication Phentermine-Topiramate Extended Release and matching placebo will be provide by Vivus, Inc (California, US). See FDA drug information package.

# Pregnancy Testing

Urine pregnancy testing will be done at visits 1, 2, 3, 4, 5, 6, and 8 for women of child bearing potential (WOCBP). Subjects who are WOCBP will be given urine pregnancy test kits (QuickVue+ hCG Combo Test (Quidel Corporation) or similar) along with instructions to complete at home. Subjects will be instructed to complete these home pregnancy test kits and report the results during the remote study visits with the study team. If a subject has a positive urine pregnancy test result at any time the subject will be withdrawn from the study and instructed to stop use of the study medication/placebo immediately. The subject will be given a referral to their primary care provider.

# Statistical Considerations

**Primary endpoint:** Total body weight loss at 3 months

**Secondary endpoints:**

- - - - Total body weight loss at 6, 9 and 12 months
      - number of steps (average per week at 3, 6, 9 and 12 months)
      - calories tracked: number times recorded, calories per day (average per week at 3, 6, 9 and 12 months)
      - number of exercise sessions (average per week at 3, 6, 9 and 12 months)
      - hours/week using app/tracker (average per week at 3, 6, 9 and 12 months)
      - weight loss difference in clinic vs. Bluetooth scale (weight loss difference 3, 6, 9 and 12 months)
      - Quality of life SF36 (at 3, 6, 9 and 12 months)
      - improvement in obesity-related comorbidities (diabetes/HbA1c, hypertension/SBP-DBP, hyperlipidemia/TC-LDL-HDL-Tg, Sleep apnea/CPAP, Joint Disease/pain)

**Design:** We propose a randomized, double-blinded, placebo-controlled trial of 80 participants with obesity to compare effects of Phentermine-topiramate ER vs placebo in weight loss with 1 year follow up.

**Sample size assessment and power calculation:** In our recent pilot study [with Liraglutide 3.0 mg vs. placebo], the standard deviation (SD) for the overall weight change (pre-post at12 weeks) observed was 2.8kg [36] and observed weight loss in the control/placebo group was 6.1kg.

Conservatively assuming a standard deviation of 3kg within groups,

| Difference to Detect (kg) – reflects greater weight loss in active arm vs placebo | Sample Size per group | TOTAL Sample Size | With 10% dropout at 3 months | With 15% dropout at 3 months | With 20% dropout at 3 months |
| --- | --- | --- | --- | --- | --- |
| 1kg (ex: 7.1 vs 6.1) | 143 | 286 | 318 | 338 | 358 |
| 1.5kg | 64 | 128 | 142 | 152 | 160 |
| 2 kg | 37 | 74 | 82 | 88 | 94 |

Actual power is anticipated to be higher when accounting for baseline weight using Analysis of Covariance (ANCOVA) methods.

# Statistical Analysis:

The primary analysis will be conducted under intention to treat (ITT) principles. Since study drug is administered double-blind, all subjects taking at least one dose of study drug will be included in analyses.

Primary endpoint: The primary endpoint is weight at 3 months (12 week visit), compared between groups using Analysis of Covariance (ANCOVA), adjusted for baseline weight. Subjects without follow up (dropouts) will have values imputed using multiple imputation. This approach assumes the distribution of missing data is random after conditioning on observed data in the imputation process. A secondary approach will consider complete case data, though this does not generally adhere to ITT principles.

Weight is recorded longitudinally at several post-baseline timepoints. A secondary analysis will model these longitudinal data using linear mixed effects models, adjusted for baseline weight. Additional adjustment variables will be included based on *a priori* determination by investigators that such variables may be associated with the dropout process. Thus, any dropout is assumed Missing At Random as a function of observed data. Time after randomization will be included as a discrete ordinal variable corresponding to the visit number. The primary comparison is a time by treatment group interaction, so that contrasts of the variables in the model will allow estimation of the treatment effect at each visit.

An interim analysis will be performed after 50% of subjects have completed their 3 month visit. This will allow the study team to review data quality and check assumptions related to the power calculation (including the standard deviation above). Investigators will remain blinded to group comparisons and while investigators have no intention of stopping the study early (regardless of interim results), the analysis at study completion will be conservatively adjusted using the O’Brien-Fleming boundary (two-sided significance level of 0.0492).

Secondary endpoints will be assessed similarly. Steps, calories, and hours using the app tracker will be collected by the device. These will be aggregated to a weekly total or daily average over the week-long period to smooth out day-to-day variation. Analyses will be performed using linear mixed effects models with these weekly total data, separately for each endpoint. Weight loss as recorded by the Bluetooth scale at home will be analyzed similarly, taking the average of assessments over the course of each week as the outcome [recall, the primary endpoint is weight loss measured in clinic at study visits]. Number of exercise sessions will be evaluated, also as a weekly total number of sessions, using generalized linear mixed effects models with the outcome analyzed as a Poisson count. The distribution of each outcome will be assessed and alternative approaches considered as necessary. Quality of Life questionnaires will be assessed at study visits and analyzed using linear mixed effects models to compare between groups. The primary outcome has been pre-specified and no adjustment will be made for comparisons of these secondary outcomes.

An exploratory aim will evaluate adherent vs non-adherent patients – and further the interaction between adherent (vs non-adherent) and randomized group, to assess whether there is a differential response to Phentermine-Topiramate among exercise adherent patients. Adherence will be defined by 80% use during the first 3 months.

# Anticipated results and significance:

Our study will demonstrate the importance of combining a consumer-based wearable activity tracker with an Anti-obesity Pharmacotherapy in Obesity.

# Potential pitfalls, precautions taken, and alternative strategies:

1. Feasibility - Given high volume of patients interested in weight loss, we are confident we will recruit sufficient participants for these studies that involve only noninvasive tests and standard of care treatment.
2. Statistical power has been addressed with appropriate sample sizes to demonstrate a difference in weight change vs. placebo.

# References

1 Gadde, K. M. *et al.* Effects of low-dose, controlled-release, phentermine plus topiramate combination on weight and associated comorbidities in overweight and obese adults (CONQUER): a randomised, placebo-controlled, phase 3 trial. *Lancet* **377**, 1341-1352, doi:10.1016/s0140-6736(11)60205-5 (2011).

2 Ng, M. *et al.* Global, regional, and national prevalence of overweight and obesity in children and adults during 1980-2013: a systematic analysis for the Global Burden of Disease Study 2013. *Lancet*, doi:10.1016/S0140-6736(14)60460-8 (2014).

3 Flegal, K. M., Carroll, M. D., Kit, B. K. & Ogden, C. L. Prevalence of obesity and trends in the distribution of body mass index among US adults, 1999-2010. *JAMA : the journal of the American Medical Association* **307**, 491-497, doi:10.1001/jama.2012.39 (2012).

4 Hensrud, D. D. & Klein, S. Extreme obesity: a new medical crisis in the United States. *Mayo Clin Proc* **81**, S5-10 (2006).

5 Yach, D., Stuckler, D. & Brownell, K. D. Epidemiologic and economic consequences of the global epidemics of obesity and diabetes. *Nat Med* **12**, 62-66, doi:10.1038/nm0106-62 (2006).

6 Daniels, S. R., Jacobson, M. S., McCrindle, B. W., Eckel, R. H. & Sanner, B. M. American Heart Association Childhood Obesity Research Summit: executive summary. *Circulation* **119**, 2114-2123, doi:10.1161/CIRCULATIONAHA.109.192215 (2009).

7 Low, S., Chin, M. C. & Deurenberg-Yap, M. Review on epidemic of obesity. *Ann Acad Med Singapore* **38**, 57-59 (2009).

8 Ogden, C. L., Yanovski, S. Z., Carroll, M. D. & Flegal, K. M. The epidemiology of obesity. *Gastroenterology* **132**, 2087-2102, doi:10.1053/j.gastro.2007.03.052 (2007).

9 World-Health-Organisation. *Fact sheet: obesity and overweight.* , 2012).

10 Tsai, A. G., Williamson, D. F. & Glick, H. A. Direct medical cost of overweight and obesity in the USA: a quantitative systematic review. *Obes Rev* **12**, 50-61, doi:10.1111/j.1467-789X.2009.00708.x (2011).

11 Riebe, D. *et al.* Updating ACSM's Recommendations for Exercise Preparticipation Health Screening. *Med Sci Sports Exerc* **47**, 2473-2479, doi:10.1249/MSS.0000000000000664 (2015).

12 Jensen, M. D. *et al.* 2013 AHA/ACC/TOS Guideline for the Management of Overweight and Obesity in Adults: A Report of the American College of Cardiology/American Heart Association Task Force on Practice Guidelines and The Obesity Society. *Journal of the American College of Cardiology* **63**, 2985-3023, doi:10.1016/j.jacc.2013.11.004 (2014).

13 Apovian, C. M. *et al.* Pharmacological management of obesity: an endocrine Society clinical practice guideline. *J Clin Endocrinol Metab* **100**, 342-362, doi:10.1210/jc.2014-3415 (2015).

14 Sjostrom, L. *et al.* Randomised placebo-controlled trial of orlistat for weight loss and prevention of weight regain in obese patients. European Multicentre Orlistat Study Group. *Lancet* **352**, 167-172 (1998).

15 Hollander, P. A. *et al.* Role of orlistat in the treatment of obese patients with type 2 diabetes. A 1-year randomized double-blind study. *Diabetes Care* **21**, 1288-1294 (1998).

16 Davidson, M. H. *et al.* Weight control and risk factor reduction in obese subjects treated for 2 years with orlistat: a randomized controlled trial. *JAMA* **281**, 235-242 (1999).

17 Gadde, K. M. *et al.* Effects of low-dose, controlled-release, phentermine plus topiramate combination on weight and associated comorbidities in overweight and obese adults (CONQUER): a randomised, placebo-controlled, phase 3 trial. *Lancet* **377**, 1341-1352, doi:10.1016/S0140-6736(11)60205-5 (2011).

18 Smith, S. *et al.* Multicenter, placebo-controlled trial of lorcaserin for weight management. *The New England journal of medicine* **363**, 245-256, doi:10.1056/NEJMoa0909809 (2010).

19 Apovian, C. M. *et al.* A randomized, phase 3 trial of naltrexone SR/bupropion SR on weight and obesity-related risk factors (COR-II). *Obesity* **21**, 935-943, doi:10.1002/oby.20309 (2013).

20 Pi-Sunyer, X. *et al.* A Randomized, Controlled Trial of 3.0 mg of Liraglutide in Weight Management. *New England Journal of Medicine* **373**, 11-22, doi:10.1056/Nejmoa1411892 (2015).

21 MD, R. *US Food and Drug Administration Endocrinologic and Metabolic Drugs Advisory Committee Clinical Briefing Document February 22, 2012.*, <<http://www.fda.gov/downloads/AdvisoryCommittees/CommitteesMeetingMaterials/Drugs/EndocrinologicandMetabolicDrugsAdvisoryCommittee/UCM292315.pdf>> (February 22, 2012.).

22 Garvey, W. T. *et al.* Two-year sustained weight loss and metabolic benefits with controlled-release phentermine/topiramate in obese and overweight adults (SEQUEL): a randomized, placebo-controlled, phase 3 extension study. *The American journal of clinical nutrition* **95**, 297-308, doi:10.3945/ajcn.111.024927 (2012).

23 Allison, D. B. *et al.* Controlled-release phentermine/topiramate in severely obese adults: a randomized controlled trial (EQUIP). *Obesity* **20**, 330-342, doi:10.1038/oby.2011.330 (2012).

24 Lyons, E. J., Lewis, Z. H., Mayrsohn, B. G. & Rowland, J. L. Behavior change techniques implemented in electronic lifestyle activity monitors: a systematic content analysis. *J Med Internet Res* **16**, e192, doi:10.2196/jmir.3469 (2014).

25 Preusse, K. C., Mitzner, T. L., Fausset, C. B. & Rogers, W. A. Older Adults' Acceptance of Activity Trackers. *J Appl Gerontol* **36**, 127-155, doi:10.1177/0733464815624151 (2017).

26 Cadmus-Bertram, L. A., Marcus, B. H., Patterson, R. E., Parker, B. A. & Morey, B. L. Randomized Trial of a Fitbit-Based Physical Activity Intervention for Women. *Am J Prev Med* **49**, 414-418, doi:10.1016/j.amepre.2015.01.020 (2015).

27 Skrepnik, N. *et al.* Assessing the Impact of a Novel Smartphone Application Compared With Standard Follow-Up on Mobility of Patients With Knee Osteoarthritis Following Treatment With Hylan G-F 20: A Randomized Controlled Trial. *JMIR Mhealth Uhealth* **5**, e64, doi:10.2196/mhealth.7179 (2017).

28 Brickwood, K. J., Watson, G., O'Brien, J. & Williams, A. D. Consumer-Based Wearable Activity Trackers Increase Physical Activity Participation: Systematic Review and Meta-Analysis. *JMIR Mhealth Uhealth* **7**, e11819, doi:10.2196/11819 (2019).

**Protocol Ammendment History**

- Mod 5/29/2020
  - 1) Revision of the baseline visit procedures in the protocol and consent for subjects who may have completed portions up to 3 years prior
  - 2) Removal of saliva sampling from the protocol
  - 3) Revision of screening to include virtual screening in the consent form
  - 4) Revision of the consenting process to use digital signature capture and electronic consenting
  - 5) Addition of a recruitment phone script; and 6) Addition of an electronic consent cover email.
  - The Committee noted receipt of the revised protocol, version 2.0, dated May 07, 2020, reflecting the modifications.
- Mod 7/10/2020
  - 1) Revision of the number of in-person visits in the protocol and consent form
  - 2) Change return of stool kit from Visit 2 to Visit 3 in the consent form
  - 3) Revision of Visit 4 to a Remote Study Visit in the protocol and consent form.
  - The Committee noted receipt of the revised protocol, Version 3.0 dated June 12, 2020, reflecting the modifications.
- Mod 8/12/2020
  - 1) Revision of the visit numbers to align with the consent form
  - 2) Addition of 45 minute blood draw to the protocol to align with the consent form.
  - The modification constitutes a minor change to previously approved research, and therefore was eligible for expedited review in accordance with 45CFR46.110(b)(2) & 21CFR56.110(b)(2). The Reviewer determined the modification(s) pose no more than minimal risk to subjects.
- Mod 7/9/2021
  - 1) Addition of DEXA scan and blood draw at Visit 8/End of Study; Radiation Safety and Clinical Trials Committee approval dated June 8, 2021
  - 2) Other administrative changes in protocol.
  - The Committee noted receipt of the revised protocol, Version 5.0 dated June 22, 2021, reflecting the modifications.
- No modifications were made to the statistical analysis plan.

**Protocol**

**Effect of an anti-Obesity Medication Phentermine-Topiramate Extended Release pharmacotherapy vs placebo among patients with obesity using a wearable activity trackers: Randomized, Double-blinded, Placebo-Control, 1-Year, Single-Center Trial**

**Principal Investigators:**

- Andres Acosta M.D., Ph.D., Division of Gastroenterology and Hepatology, Department of Medicine, Mayo Clinic, Rochester, MN;

**Co-Investigator:**

- Michael Camilleri, M.D., Division of Gastroenterology and Hepatology, Department of Medicine, Mayo Clinic, Rochester, MN
- Donald Hensrud, M.D., Division of General Internal Medicine, Department of Medicine, Mayo Clinic, Rochester, MN
- Phillip Schulte, Ph.D., Division of Biomedical Statistics & Informatics, Department of Health Sciences Research, Mayo Clinic, Rochester, MN.
- Duane Burton, M.S. Division of Gastroenterology and Hepatology, Department of Medicine, Mayo Clinic, Rochester, MN

**Coordinator:**

- Bill Rossini, Division of Gastroenterology and Hepatology, Department of Medicine, Mayo Clinic, Rochester, MN
- Irene Busciglio, B.S. Division of Gastroenterology and Hepatology, Department of Medicine, Mayo Clinic, Rochester, MN

**Research Fellows:**

- Daniel Gonzalez-Izundegui, M.D., Division of Gastroenterology and Hepatology, Department of Medicine, Mayo Clinic, Rochester, MN
- Sneha Singh M.D. Division of Gastroenterology and Hepatology, Department of Medicine, Mayo Clinic, Rochester, MN

**IRB approved:** Pending

**Conflict of Interest:** none

Version 1.0

4 February 2020

# Abstract:

**Introduction:** Obesity prevalence continues to increase worldwide. Estimated costs to the healthcare system are more than $220 billion annually. Obesity severity is associated with higher cardiovascular mortality. FDA-approved medications, devices and surgeries have shown long-term improvements in obesity and diabetes, however, their use (population penetrance) remains low. On the contrary, and despite of contradicting published literature, numerous wearable technologies specific to physical activity and diet are widely adopted with minimal long-term data or benefits. Little is known about the effect anti-obesity pharmacotherapy among patients undergoing lifestyle intervention that includes a consumer-based wearable activity tracker.

**Hypothesis and Aims:** We hypothesize that anti-obesity pharmacotherapy (vs placebo) will have an effect on weight loss among patients using an activity tracker as part of a lifestyle intervention. Thus, we aimed to study in a randomized, double blinded, placebo-control, 1-year, single-center trial the effect of Phentermine-topiramate ER (Anti-obesity Pharmacotherapy) vs placebo among patients with obesity using a wearable activity tracker as part of standard lifestyle intervention.

**Methods/Study Design:** We propose a randomized, single-center trial in 80 patients with obesity to study the Effect of **an** anti-Obesity Medication Phentermine-Topiramate Extended Release vs placebo among patients using a wearable activity tracker in weight loss and obesity related comorbidities in 12 months. All the participants will receive a wearable activity tracker with a Bluetooth scale as part of an intense lifestyle intervention (14 visits total with dietitians, physicians and behavioral therapists (or 6 visits and at least one contact per week virtually with wellness coach) and they will be randomized 1:1 to placebo or Phentermine-topiramate ER (Dosing of 3.75/23 mg daily for 15 days, increased to 7.5/46 mg daily). Participants will be randomized according to a computer generated randomization schedule generated by the study statistician’s office and submitted to the Mayo Clinic CTSA research pharmacy. Allocation will be concealed. Study end-points: a) Primary: Total body weight loss at 3 months among the groups; and b) secondary: Total body weight loss at 6, 9 and 12 months among the groups; number of steps (average per week at 3, 6, 9 and 12 months among the groups); calories tracked: number times recorded, calories per day (average per week at 3, 6, 9 and 12 months among the groups), number of exercise sessions (average per week at 3, 6, 9 and 12 months among the groups); hours/week using app/tracker (average per week at 3, 6, 9 and 12 months among the groups); weight loss difference in clinic vs. Bluetooth scale (weight loss difference 3, 6, 9 and 12 months); Quality of life SF36 (at 3, 6, 9 and 12 months among the groups); improvement in obesity-related comorbidities (diabetes/HbA1c, hypertension/SBP-DBP, hyperlipidemia/TC-LDL-HDL-Tg, Sleep apnea/CPAP, Joint Disease/pain).

**Protocol**

**Effect of a Consumer-Based Wearable Activity Tracker in Combination with an Anti-Obesity Pharmacotherapy in Obesity: Randomized, Placebo-Control, 1-Year, Single-Center Trial**

Table of Contents

[Abstract: 2](#_Toc26799503)

[Introduction: 4](#_Toc26799504)

[Treatment for obesity: 4](#_Toc26799505)

[Pharmacotherapy 5](#_Toc26799506)

[Phentermine-Topiramate Extended Release: 5](#_Toc26799507)

[Wearable Activity Trackers 6](#_Toc26799508)

[Hypothesis and Aims: 8](#_Toc26799509)

[Methods/Study Design: 8](#_Toc26799510)

[Randomization and Allocation 12](#_Toc26799511)

[Selection Participants 12](#_Toc26799512)

[Anthropometrics and Metabolic Characteristics studies 13](#_Toc26799513)

[Intense Lifestyle Intervention and Behavioral Treatment 14](#_Toc26799514)

[Statistical Considerations 15](#_Toc26799515)

[Statistical Analysis: 16](#_Toc26799516)

[Anticipated results and significance: 16](#_Toc26799517)

[Potential pitfalls, precautions taken, and alternative strategies: 17](#_Toc26799518)

[References 19](#_Toc26799519)

# Introduction:

Obesity prevalence continues to increase worldwide[2] and, in the United States, 69% of adults are overweight or with obesity[3]. Estimated costs to the healthcare system are more than $480 billion annually. Increased severity of obesity correlates with a higher prevalence of the associated co-morbidities. Likewise, obesity increases the risk of premature mortality [4]. Obesity affects almost every organ system in the body and increases the risk of numerous diseases including type 2 diabetes mellitus, hypertension, dyslipidemia, cardiovascular disease, and cancer. It is estimated that a man in his twenties with a BMI over 45 will have a 22% reduction (13 years) in life expectancy.

***Obesity*** is defined as the amount of excess of adipose tissue at which health risks increase[5]. Normal weight, overweight and obesity can be measured by Body Mass Index (BMI). BMI is calculated by weight (kg) divided by the square of the height (m^2^). The BMI for an adult healthy weight is from 18.5 to 24.9 kg/m^2^, overweight is from 25 to 29.9 kg/m^2^ and obese is 30 kg/m^2^ or above. Obesity is considered morbid or severe when BMI is higher than 40 kg/m^2^ (Guidelines 1998). In children, obesity is measured as BMI higher than the 95^th^ percentile related to their age and sex [6, 7]. Obesity can also be measured by waist circumference defined as larger than 102 cm in men and 88 cm in women. BMI and waist circumference are associated with health risks and obesity–related co-morbidities.

**The Obesity Epidemic**: Obesity has reached epidemic proportions in developed countries and its prevalence is increasing in developing countries [7]. In the United States of America (USA), the prevalence of overweight adults is 64%, and obese adults is 30.5% [8]. In children and adolescents, the obesity prevalence has increased to 17.1% [6]. The World Health Organization indicated that globally, in the year 2005, there were approximately 1.6 billion overweight adults, and of those, 400 million were obese [9]^.^ This alarming obesity epidemic poses a heavy burden to the U.S. economy, costing more than $150 billion every year—10 percent of the total health budget—according to the Centers for Disease Control and Prevention (CDC 2012). In 2009, the U.S. Agency for Healthcare Research and Quality reported that overweight and obese people spend 43 percent more each year in medical expenses than those who have normal weight. The Agency also found that obese workers are paid less than their coworkers who have normal weight [10].

## Treatment for obesity:

The 2013 Obesity Guidelines suggest that to achieve weight loss, an energy deficit is essential. Reducing dietary energy intake below that required for energy balance can be achieved through a reduction of daily calories to 1200-1500 for women, and 1,500-1800 for men (kilocalorie levels are usually adjusted for the individual’s body weight and physical activity levels); or estimation of individual daily energy requirements and prescription of an energy deficit of 500 kcal/d or 750 kcal/d. Recommendations for young children through adolescence vary in order to support normal growth and development occurring during these years. The Academy of Nutrition and Dietetics Evidence Analysis Library recommends no fewer than 900 kcal/day for 6-12 year olds who are medically monitored and no fewer than 1200 kcal/day for 13-18 year olds (Academy of Nutrition and Dietetics Weight Management Position Paper which provides an overview of a nutrition assessment: http://www.eatrightpro.org/resource/practice/position-and-practice-papers/position-papers/weight-management). Evidence supports greatest long-term success with an individualized, structured meal plan in place. A registered dietitian nutritionist can play an important role in designing the nutrition intervention tailored to address each patient’s unique needs and circumstances, taking into consideration factors such as insulin resistance. Any diet program that meets this required energy deficit is appropriate to adopt, and comparative trials have shown no long-term superiority between different macronutrient composition or elimination diets. Furthermore, it is important to adhere to a balanced diet that provides a variety of items from all food groups and limits potentially harmful food ingredients like added sugars, sodium and alcohol. Additionally, guidelines recommend limiting or avoiding liquid calories (i.e. sodas, juices, alcohol, etc.). And, finally, the meal plan should be designed in such a way that the individual is likely to follow it.

Along with the prescription for a reduced calorie diet, a comprehensive lifestyle intervention program should prescribe increased aerobic physical activity (such as brisk walking) for ≥150 min/week (equal to ≥30 min/d most days of the week), and a goal of >10,000 steps per day. Higher levels of physical activity, approximately 200 to 300 min/wk., are recommended to maintain the weight lost or minimize weight regain in the long term (>1 year) [11]. The diet and physical activity can be in combination with a hospital/university or commercial behavior program; these are comprehensive lifestyle interventions that usually provide structured behavior strategies to facilitate adherence to diet and activity recommendations. These strategies include regular self-monitoring of food intake, body weight, physical activity, and food cravings. These same behaviors are recommended to maintain lost weight, with the addition of frequent (i.e., weekly or more frequent) monitoring of body weight[12].

## Pharmacotherapy

In addition to diet, exercise and behavioral modification, pharmacotherapies should be considered as an adjunct to lifestyle changes in patients who have been unable to lose and maintain weight with diet and exercise alone. They should also be considered in people whose history or clinical circumstances require expedited weight loss. Medication should not be used alone, but in combination with an intensive lifestyle program.

Pharmacotherapy for the treatment of obesity can be considered if a patient has a body mass index (BMI) ≥ 30 kg/m^2^ or a BMI ≥ 27 kg/m^2^ with weight-related co-morbidities such as hypertension, type 2 diabetes, dyslipidemia and obstructive sleep apnea[12]. Medical therapy should be initiated with dose escalation based on efficacy and tolerability to the recommended dose. An assessment of efficacy and safety at least monthly for the first three months and then at least every three months. In patients who have cardiovascular disease, guidelines recommend against prescribing sympathomimetic agents such as phentermine and phentermine/topiramate extended release (ER). Lorcaserin and orlistat are safer alternatives. In patients with T2DM, the guidelines suggest antidiabetic agents that promote weight loss such as glucagon-like peptide (GLP-1) analogs which reduce hyperglycemia in addition to the first-line agent for T2DM, metformin[13].

| **Medication / dose** | **Clinical data** | **Mean weight change from baseline after 1 year** | **Weight loss after 1 year (Proportion of participants)** | | | **References** |
| --- | --- | --- | --- | --- | --- | --- |
|  |  |  | **>5%** | **>10%** | **>15%** |  |
| Orlistat  120 mg TID | Clinical data from three trials | −6.0 to 10.3  Kg vs −2.6 to 6.1 Kg with placebo | **36–67%**  (vs.16–43.6%) | **17 - 38.9**  (vs. 8.8 – 24.8) | **NA** | [14-16] |
| Phentermine/ topiramate ER 15 mg/92 mg QD | 1‐year trial, people with obesity (BMI ≥35 kg/m^2^) | −10.9% vs −1.6% with placebo | **70%**  (vs.21%) | **48%**  (vs. 7%) | NA | [17] |
| Lorcaserin  10 mg BID | 2‐year trial, people with obesity or overweight and ≥1 comorbidity | −5.8% vs −2.5% with placebo | **47%**  (vs. 23%) | **22.6**  (vs. 7.7) | NA | [18] |
| Naltrexone/ bupropion SR  32 mg/360 mg | Four 56‐week trials, people with obesity and ≥1 comorbidity | −5.4% vs −1.3% with placebo (COR‐I) | **42%**  (vs. 17%) | **28.3**  (vs. 5.7) | **13.5**  (vs. 2.4) | [19] |
| Liraglutide  3.0 mg QD | 56‐week trial, people with obesity or overweight and ≥1 comorbidity | −7.4% vs −3.0% with placebo | **62%**  (vs. 34%) | **33.1%** (vs. 10.6%) | **14.4%**  (vs. 3.5%) | [20] |

Phentermine-Topiramate Extended Release: When low-dose, controlled-release, phentermine was combined with the glutamatergic and GABA-ergic antiepileptic topiramate in a large phase III study (more than 1400 participants on treatment arms with different doses), subjects lost 10.2 kg on 15/92 mg combination therapy vs. 1.4 kg on placebo over 56 weeks [17]. The most common adverse events were dry mouth, paresthesias, constipation, insomnia, dizziness, and dysgeusia. Depression- and anxiety-related adverse events were also observed. The medication had favorable effects on glycemia, including prevent progression to diabetes, improvements in lipids, blood pressure, sleep apnea, and quality of life measures. There was also, as previously noted, a small but consistent increase in pulse rate [21]. The overall rate of adverse effects decreased in weeks 56–108 compared to weeks 0–56; among which dry mouth, constipation and paresthesias were the most prevalent There were 19 pregnancies carried to term during these studies none of which resulted in congenital abnormalities [21-23].


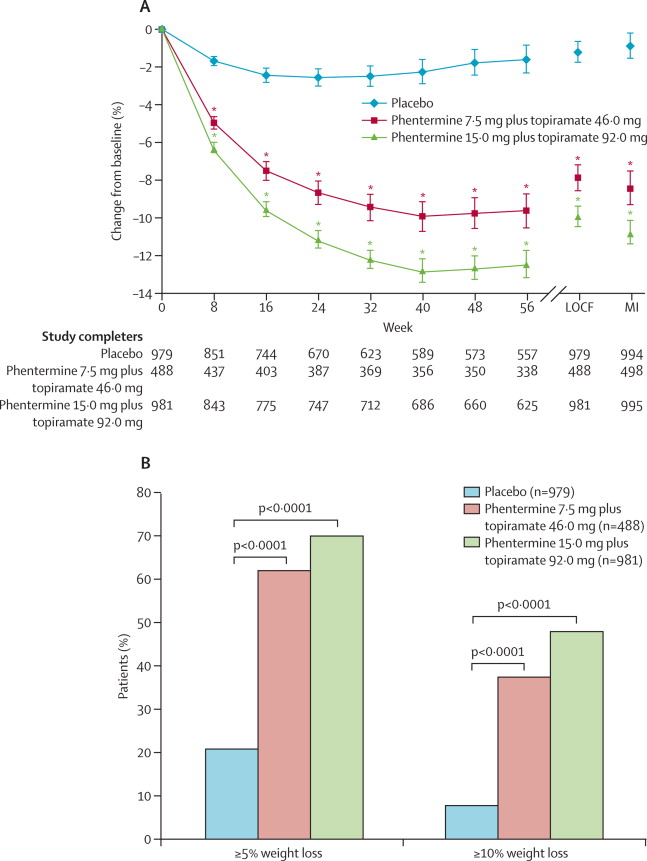


Figure 1. Effects of phentermine plus topiramate on bodyweight. Patients with at least 5% and at least 10% weight loss. [1]

In July 2012, the FDA voted for approval of phentermine (3.75–15mg/d) plus extended release topiramate (23–92mg/d) as an adjunct to diet and physical activity for treatment of obesity among adult individuals with BMI≥30kg/m^2^ or BMI≥27kg/m^2^ with at least one obesity-related comorbid condition. The drug will carry a warning of potential increased risk for orofacial clefts in neonates exposed to topiramate during the first trimester of gestation and will be subject to a Risk Evaluation and Mitigation Strategy (REMS) that will restrict prescribing to trained clinicians, will require effective contraception and monthly pregnancy tests for reproductive age women, and will restrict dispensing to specific mail-order pharmacies. The company is also required to carry a long-term cardiovascular outcomes trial. No randomized pediatric studies have as yet been reported. Noteworthy, the high dose of PhenTop was associated with a mean weight loss of 9.8%; however, only 48% of patients lost >10% of their body weight, and 30% of patients lost <5% of their body weight (Figure 1).

## Wearable Activity Trackers

Consumer-based wearable activity trackers are now readily available and can provide individuals with the ability to objectively monitor their physical activity levels. In addition, when combined with the use of smartphone and computer apps, they may assist users through a range of motivational and tracking tools to better manage their personal health[24]. In addition to providing real-time feedback relating to daily steps and energy expenditure, consumer-based wearable activity trackers have the potential to provide specific, tailored feedback through specifically designed algorithms or by health professionals. This type of emerging technology may provide an alternative means of providing ongoing support and motivation to individuals both looking to increase their activity levels or to maintain activity levels following a structured lifestyle intervention[25]. Moreover, consumer-based wearable activity trackers may assist in reducing the resource and time burden associated with traditional methods of providing ongoing support. Randomized controlled trials have shown that these devices have promise in relation to increasing physical activity participation[26, 27]; however, participant numbers in individual studies tend to be low, making it difficult to adequately assess the benefits of these devices. Furthermore, there is limited research relating to their long-term adherence and effectiveness.

A recent systematic review[28] of that aimed to determine the effects of interventions utilizing consumer-based wearable activity trackers on physical activity participation and sedentary behavior when compared with interventions that do not utilize activity tracker feedback, showed that there was a significant increase in daily step count (standardized mean difference [SMD] 0.24; 95% CI 0.16 to 0.33; P<.001), moderate and vigorous physical activity (SMD 0.27; 95% CI 0.15 to 0.39; P<.001), and energy expenditure (SMD 0.28; 95% CI 0.03 to 0.54; P=.03) and a nonsignificant decrease in sedentary behavior (SMD −0.20; 95% CI −0.43 to 0.03; P=.08) following the intervention versus control comparator across all studies in the meta-analyses. In general, included studies were at low risk of bias, except for performance bias. Heterogeneity varied across the included meta-analyses ranging from low (I2=3%) for daily step count through to high (I2=67%) for sedentary behavior. Utilizing a consumer-based wearable activity tracker as either the primary component of an intervention or as part of a broader physical activity intervention has the potential to increase physical activity participation. As the effects of physical activity interventions are often short term, the inclusion of a consumer-based wearable activity tracker may provide an effective tool to assist health professionals to provide ongoing monitoring and support[28].

Online support system: VitalCare (VitalTech Affiliates LLC) is a digital health platform that allows remote collection of date from the wearable tracker and digital wellness devices used as part of this study. It also will allow subjects to document study medication compliance and will allow the remote visits to be conducted through a video conference between subjects and appropriate study team members.

FDA-approved medications, devices and surgeries have shown long-term improvements in obesity and diabetes, however, their use (population penetrance) remains low (less than 1% market use). On the contrary, and despite of contradicting published literature, numerous wearable technologies specific to physical activity and diet are widely adopted with minimal long-term data or benefits. Little is known about the effect anti-obesity pharmacotherapy among patients undergoing lifestyle intervention that includes a consumer-based wearable activity tracker.

# Hypothesis and Aims:

We hypothesize that anti-obesity pharmacotherapy (vs placebo) will have an effect on weight loss among patients using an activity tracker as part of a lifestyle intervention. Thus, we aimed to study in a randomized, double-blinded, placebo-control, 1-year, single-center trial the effect of Phentermine-topiramate ER (Anti-obesity Pharmacotherapy) vs placebo among patients with obesity using a wearable activity tracker as part of standard lifestyle intervention.

# Methods/Study Design:

We propose a randomized, double blinded, single-center trial in 80 patients with obesity to study the Effect of an anti-Obesity Medication Phentermine-Topiramate Extended Release vs placebo among patients using a wearable activity trackers in weight loss and obesity related comorbidities in 12 months. All the participants will receive a wearable activity tracker and digital wellness devices (Bluetooth scale, Bluetooth pulse oximeter and Bluetooth blood pressure monitor) as part of an intense lifestyle intervention (9 in person visits total with dietitians, physicians and other study team members. At least one contact per month virtually with a member of the study team when an in person visit is not scheduled. Subjects will be randomized 1:1 to placebo or Phentermine-topiramate ER (Dosing of 3.75/23 mg daily for 15 days, increased to 7.5/46 mg daily). Participants will be randomized according to a computer generated randomization schedule generated by the study statistician’s office and submitted to the Mayo Clinic CTSA research pharmacy. Allocation will be concealed. Study end-points: a) Primary: Total body weight loss at 3 months among the groups; and b) secondary: Total body weight loss at 6, 9 and 12 months among the groups; number of steps (average per week at 3, 6, 9 and 12 months among the groups); calories tracked: number times recorded, calories per day (average per week at 3, 6, 9 and 12 months among the groups), number of exercise sessions (average per week at 3, 6, 9 and 12 months among the groups); hours/week using app/tracker (average per week at 3, 6, 9 and 12 months among the groups); weight loss difference in clinic vs. Bluetooth scale (weight loss difference 3, 6, 9 and 12 months); Quality of life SF36 (at 3, 6, 9 and 12 months among the groups); improvement in obesity-related comorbidities (diabetes/HbA1c, hypertension/SBP-DBP, hyperlipidemia/TC-LDL-HDL-Tg, Sleep apnea/CPAP, Joint Disease/pain).

Study flowsheet:

Recruitment 100 subjects

Randomization 80 subjects

Digital support + Placebo n = 40

Digital Support + Phentermine-Topiramate ER n = 40

Visit schedule:

1. Visit 1-Screening visit
2. Visit 2-Baseline anthroprometric and metabolic studies
3. Visit 3-Counseling / Randomization / wearable tracker, digital wellness devices and Medication assignment and disbursement.
4. Visit 4-2-week follow up (Remote visit)
5. Visit 5-4-week follow up
6. Remote visits-2 month
7. Visit 6-3 month follow up / samples collection / body composition
8. Remote visit-4 month
9. Remote visit- 5 month
10. Visit 7- 6 month follow up
11. Remote visit-7 month
12. Remote visit-8 month
13. Visit 8-9 monthfollow up
14. Remote visit-10 month
15. Remote visit-11 month
16. Visit 9-12 month End of study

|  |  |  |  | Months | | | | | | | | | | | | |
| --- | --- | --- | --- | --- | --- | --- | --- | --- | --- | --- | --- | --- | --- | --- | --- | --- |
|  |  |  |  | 1 | | 2 | 3 | 4 | 5 | 6 | 7 | 8 | 9 | 10 | 11 | 12 |
| Study Procedures | Visit 1 Screening | Visit 2 Baseline Studies | Visit 3  Counseling and Randomization | Visit 4  2-Week  (+/- 3 days) | Visit 5  4-Week  (+/- 3 days) | Remote Study Visits | Visit 6 | Remote Study Visits | Remote Study Visits | Visit 7 | Remote Study Visits | Remote Study Visits | Visit 8 | Remote Study Visits | Remote Study Visits | Visit 9 |
| Informed Consent | X |  |  |  |  |  |  |  |  |  |  |  |  |  |  |  |
| Medical History and Physical Examination | X |  |  |  |  |  |  |  |  |  |  |  |  |  |  |  |
| Pregnancy Test | X* | X* | X* |  | X* |  | X* |  |  | X* |  |  | X* |  |  | X* |
| Vital Signs | X | X | X | X | X |  | X |  |  | X |  |  | X |  |  | X |
| Metabolic Studies |  | X |  |  |  |  |  |  |  |  |  |  |  |  |  |  |
| Blood Collection |  | X |  |  |  |  | X** |  |  | X** |  |  | X** |  |  | X** |
| Medication Diary |  |  |  |  |  |  |  |  |  |  |  |  |  |  |  |  |
| Dispense medication |  |  | X |  |  |  | x |  |  | x |  |  | x |  |  |  |
| Medication Reconciliation |  |  | X |  |  |  | X |  |  | X |  |  | X |  |  |  |
| Wearable Tracker and Digital Wellness Devices Given |  |  | X |  |  |  |  |  |  |  |  |  |  |  |  |  |
| Randomization and Medication/Placebo Prescription |  |  | X |  |  |  |  |  |  |  |  |  |  |  |  |  |
| Behavioral Questionnaires | X*** | X^a^ |  |  |  |  |  |  |  |  |  |  |  |  |  | X |
| Stool Sample |  | X |  |  |  |  |  |  |  |  |  |  |  |  |  |  |
| Adverse Event Assessment |  |  |  | X | X | X | X | X | X | X | X | X | X | X | X | X |
| Review VitalCare Measurments/pregnancy test results |  |  |  | X | X | X | X | X | X | X | X | X | X | X | X | X |
| Lifestyle Intervention Review |  |  | X |  |  |  |  |  |  |  |  |  |  |  |  |  |
| Home Urine Pregnancy Tests Given |  |  |  |  | X |  | X |  |  | X |  |  | X |  |  |  |

* may be done up to 48 hours prior to visit.

**fasting blood draw only (basic metabolic panel, lipid panel, HbA1C, hsCRP, plasma hormones and proteomics.

***HADS, AUDIT-C and Eating disorders questionnaire only

^a^ All questionnaires except HADS, AUDIT-C and Eating disorders questionnaire

^b^ Visit window for visits 6, 7, 8, 9 and all remote visit is +/- 5 days.

## Randomization and Allocation

A computer generated randomization schedule generated by the study statistician’s office will be submitted to the Mayo Clinic CCaTS Research Pharmacy. Randomization will be based on guiding pharmacotherapy or placebo. Allocations will be concealed. This study will be blinded until data are transmitted to the statistician for data lock. All subjects will be given a verbal explanation of the study, provided time to read and study the written consent form and its information, given opportunities to ask questions and a copy of the consent form. Participants will be informed of their right to withdraw from the study at any time without prejudice to their clinical management now or in the future. Consent will be sought by one of the medical doctor investigators or the study coordinator, and consent will be documented by the participant’s signature on the consent form. Mayo’s Institutional Review Board will approve the process and protocol. All the members of multidisciplinary team for weight management (i.e. physicians, coordinators, clinical assistants, registered dietitians will remain blinded).

If unblinding is needed for subject safety the Principal Investigator or a Co-Investigator will contact a research Pharmacist or the research pharmacy manager and provide documentation of reason for study unblinding and the subject will be withdrawn from the study.  The study team will document and report the reason for unblinding as required by the Mayo Clinic IRB.

## Selection Participants

We plan to study a cohort of 80 patients with obesity (BMI>30 kg/m^2^). Participants will be recruited from the Mayo Clinic Weight Management and Nutrition Clinic, media advertising, classified ads, and existing databases of patients with obesity, including the phenome registry (Mayo Clinic IRB number 19-000030), the Mayo Clinic biobank and the right-10k cohort.

Inclusion criteria

1. Adults with obesity (BMI >30Kg/m^2^); these will be otherwise healthy individuals with no unstable psychiatric disease and controlled comorbidities or other diseases.
2. Age: 18-75 years.
3. Gender: Men or women. Women of childbearing potential will have negative pregnancy tests within 48 hours of enrollment.
4. Women of childbearing potential must agree to use a method of effective contraception during study participation.
5. Subject must have an Apple iPhone 6s or later with iOS 13 or later and be willing to download the VitalCare (VitalTech Affiliates LLC) application from the Apple App Store.
6. Able to provide written informed consent prior to any study procedures, and be willing and able to comply with study procedures

Exclusion criteria

1. History of Abdominal bariatric surgery
2. Weight is greater than 450 lbs (204 kg)
3. Recent use (within the last three months) of any antiobesity medication
4. Recent weight change (gain or loss weight greater than 3% TBW in the last 3 months)
5. Positive history of chronic gastrointestinal diseases, or systemic disease that could affect gastrointestinal motility, or use of medications that may alter gastrointestinal motility, appetite or absorption, e.g., orlistat, within the last 6 months.
6. Significant untreated psychiatric dysfunction based upon screening with the Hospital Anxiety and Depression Inventory (HAD), and the Questionnaire on Eating and Weight Patterns (binge eating disorders and bulimia). If such a dysfunction is identified by an anxiety or depression score >11 or difficulties with substance or eating disorders, the participant will be excluded and given a referral letter to his/her primary care doctor for further appraisal and follow-up.
7. Hypersensitivity or contraindication to the study medication.
8. Participant unable or unwilling to follow protocol including use of the wearable activity tracker, digital wellness devices, VitalCare application, or unwilling to sign consent.
9. Principal Investigator discretion

## Anthropometrics and Metabolic Characteristics studies

Anthropometrics Measurements: will be taken of height, weight, blood pressure, pulse, waist and hip ratio, respiration rate and temperature at screening, baseline, randomization day and visit 4, 5, 6, 7, 8 and 9.

Baseline Characteristic studies:

All participants will complete the baseline assessment at the Mayo Clinic after an 8‑hour fasting period, and the following characteristics will be measured at baseline: Fasting blood collection, body composition, resting energy expenditure, gastric emptying with meal for breakfast, behavioral questionnaires, exercise capacity and performance and buffet meal test for lunch. Blood will be collected for assessment of metabolomic biomarkers, gastrointestinal hormones, DNA (blood). Stool samples for microbiome and bile acid. Participants will return to the CRTU to pick up medication based on the randomization, the wearable activity tracker and the digital wellness devices.

Methods of metabolic studies

1. Body composition will be measured by DEXA (dual energy x-ray absorptiometry).
2. Resting energy expenditure was assessed by indirect calorimetry with a ventilated hood (Parvo Medics, Sandy, UT).
3. Gastric emptying (GE) of solids by scintigraphy: The primary endpoint is gastric half-emptying time (GE t_1/2_) [29-31]. Images will be acquired at 0, 60, 120 and 240 minutes following the normal clinical gastric emptying testing protocol without a push meal.
4. Appetite (hunger level) by visual analog score fasting and after standard meal for GE and prior to the Satiation test [30].
5. Satiation will be measure by *ad-libitum* buffet meal to measure total caloric intake and macronutrient distribution in the chosen food. Satiation will be reported in calories consumed at fullness (satiation) [30].
6. Satiety by visual analog score postprandial after standard meal for GE and after to the *ad*-libitum buffet meal test for every 30 minutes for 2 hours [30]. Satiety will be measured in length of time of fullness.
7. Samples collection, handling and storage: Samples will be collected after an overnight fast (of at least 8 hours) in the morning. Plasma will be preserved following standard guidelines and protein degradation inhibitors, kalikrein and DPP-IV inhibitors will be added to preserve the samples. Samples will be stored at -80ºC in the PI’s laboratory. Fasting samples will be collected and measure on subsequent visits (every 3 months).
   1. Fasting Blood will be collected for basic metabolic panel, lipid panel, HbA1C, hsCRP
   2. Plasma hormones and proteomics by radioimmunoassay and/or mass spectrometry measured fasting, and postprandial 15 and 90 minutes, with the primary endpoint being the peak postprandial level (test should be done simultaneously to GE).
   3. Targeted Metabolomics: We will perform quantitative, targeted metabolomics of salient classes of compounds in plasma and saliva samples using mass spectrometry.
   4. Blood DNA for genome wide association studies (GWAS)
   5. Stool will be collected and stored to study microbiome, short chain fatty acids and bile acids.
8. Self-administered questionnaires assessing affect, physical activity levels, attitudes, body image, diet, and eating behavior; details of each questionnaire are provided below. Participants will complete a series of questionnaires.
9. Hospital Anxiety and Depression Scale: HADS will be used to screen for severe anxiety or depression.
10. AUDIT-C Alcoholism Screening Test [32] - The AUDIT-C is a 3-item alcohol screening questionnaire that reliably identifies participants who are hazardous alcohol drinkers or have active alcohol use disorders. This score will be used in screening by the study physician/nurse coordinator. The AUDIT-C is scored on a scale of 0-12. Each AUDIT-C question has 5 answer choices. Points allotted are: a=0 points; b=1 point; c=2 points; d=3 points; e=4 points. In men, a score of 4 or more is considered positive, optimal for identifying hazardous drinking or active alcohol use disorders. In women, a score of 3 or more is considered positive (same as above).
11. Eating Disorders Questionnaire - The Questionnaire on Eating and Weight Patterns-Revised [33], is a valid measure of screening for eating disorders which has been used in several national multi-site field trials. Respondents are classified as binge eating disorder, purging bulimia nervosa, non-purging bulimia nervosa, or anorexia nervosa. We have used this instrument to screen for eating disorders in obese populations.
12. Three Factor eating questionnaire is 21-item questionnaire, validated, to assess for emotional eating disorders and food cravings.
13. Physical Activity Level - The four-item Physical Activity Stages of Change Questionnaire [34]will be utilized to assess the physical activity level of participants. Mayo Clinic investigators, led by co-investigator Dr. Clark, have used these items to explore the relationship between quality of life and physical activity in an NCI-funded study on long-term lung cancer survivors [34].
14. Exercise behavior*-* The Exercise Regulations Questionnaire (BREQ-3)*[35]* and its subsequent modifications have become the most widely used measures of the continuum of behavioural regulation in exercise psychology research. It has been used either as a multidimensional instrument giving separate scores for each subscale, or as a unidimensional index of the *degree* of self-determination.
15. SF-12v2- a common questionnaire used to measure quality of life
16. STOP-BANG- questionnaire used to screen for obstructive sleep apnea
17. WOMAC- questionnaire used to assess osteoarthritis of the knee and hip

### Intense Lifestyle Intervention and Behavioral Treatment

All the participants will meet the multidisciplinary team which consists of an Obesity Expert physician, registered dietitian nutritionist as standard of care in our clinical practice. All participants will guided to 1) Nutrition: Reduce dietary intake below that required for energy balance by consuming 1200calories per day for women and 1400calories per day for men; 2) Physical Activity: reach the goal of 10,000 steps or more per day; 3) Exercise: reach the goal of 150 minutes or more of cardiovascular exercise/week; 4) Limit consumption of liquid calories (i.e. sodas, juices, alcohol, etc.). All participants will receive a personal fitness tracker, where their activity and calories will be tracked. This information will be given in a booklet format.

**Wearable tracker**:

Subjects in the study will be provided with an Apple watch Series 5. The Apple watch will be connected by Bluetooth technology to the subject’s personal Apple iPhone. Subjects will be required to use the watch during the study.

**Digital Wellness Devices**:

Subjects in the study will be provided and a wireless scale, automated blood pressure cuff and pulse oximeter. These digital wellness devices will be connected by Bluetooth technology to the subject’s personal Apple iPhone.

Subjects will be allowed to keep the wearable activity tracker and the digital wellness devices if they complete the study. Subjects who withdraw from the study or are withdrawn from the study will be asked to return the digital wellness devices. Subjects will also be provided with a backpack to transport the wellness devices.

**Virtual Care:**

As part of the study subjects will be asked to download the VitalCare (VitalTech Affiliates LLC) application to the subject’s personal Apple iPhone from the Apple App Store. This application will allow the study team to conduct the remote study visits. The application will also be used to monitor subject compliance with taking the study medication and to send reminders to the subject to take the study medication. Additionally this application will record the measurements collected by the digital wellness devices given to the subjects as part of the study. The study subjects will not be required to use the digital wellness devices at any set time points, but it will be encouraged. The digital wellness devices will be used at the subject’s discretion. Any measurements recorded by the digital wellness devices will only be reviewed by the study team during the subject’s study visits, either in person visits or remote visits. Study team will not monitor the use or results of this study until the next schedule visit or until the participant brings it to our attention.

**Medication**

Medication Phentermine-Topiramate Extended Release and matching placebo will be provide by Vivus, Inc (California, US). See FDA drug information package.

# Pregnancy Testing

Urine pregnancy testing will be done at visits 1, 2, 3, 5, 6, 7, 8 and 9 for women of child bearing potential (WOCBP). Subjects who are WOCBP will be given urine pregnancy test kits (QuickVue+ hCG Combo Test (Quidel Corporation) or similar) along with instructions to complete at home. Subjects will be instructed to complete these home pregnancy test kits and report the results during the remote study visits with the study team. If a subject has a positive urine pregnancy test result at any time the subject will be withdrawn from the study and instructed to stop use of the study medication/placebo immediately. The subject will be given a referral to their primary care provider.

# Statistical Considerations

**Primary endpoint:** Total body weight loss at 3 months

**Secondary endpoints:**

- - - - Total body weight loss at 6, 9 and 12 months
      - number of steps (average per week at 3, 6, 9 and 12 months)
      - calories tracked: number times recorded, calories per day (average per week at 3, 6, 9 and 12 months)
      - number of exercise sessions (average per week at 3, 6, 9 and 12 months)
      - hours/week using app/tracker (average per week at 3, 6, 9 and 12 months)
      - weight loss difference in clinic vs. Bluetooth scale (weight loss difference 3, 6, 9 and 12 months)
      - Quality of life SF36 (at 3, 6, 9 and 12 months)
      - improvement in obesity-related comorbidities (diabetes/HbA1c, hypertension/SBP-DBP, hyperlipidemia/TC-LDL-HDL-Tg, Sleep apnea/CPAP, Joint Disease/pain)

**Design:** We propose a randomized, double-blinded, placebo-controlled trial of 80 participants with obesity to compare effects of Phentermine-topiramate ER vs placebo in weight loss with 1 year follow up.

**Sample size assessment and power calculation:** In our recent pilot study [with Liraglutide 3.0 mg vs. placebo], the standard deviation (SD) for the overall weight change (pre-post at12 weeks) observed was 2.8kg [36] and observed weight loss in the control/placebo group was 6.1kg.

Conservatively assuming a standard deviation of 3kg within groups,

| Difference to Detect (kg) – reflects greater weight loss in active arm vs placebo | Sample Size per group | TOTAL Sample Size | With 10% dropout at 3 months | With 15% dropout at 3 months | With 20% dropout at 3 months |
| --- | --- | --- | --- | --- | --- |
| 1kg (ex: 7.1 vs 6.1) | 143 | 286 | 318 | 338 | 358 |
| 1.5kg | 64 | 128 | 142 | 152 | 160 |
| 2 kg | 37 | 74 | 82 | 88 | 94 |

Actual power is anticipated to be higher when accounting for baseline weight using Analysis of Covariance (ANCOVA) methods.

# Statistical Analysis:

The primary analysis will be conducted under intention to treat (ITT) principles. Since study drug is administered double-blind, all subjects taking at least one dose of study drug will be included in analyses.

Primary endpoint: The primary endpoint is weight at 3 months (12 week visit), compared between groups using Analysis of Covariance (ANCOVA), adjusted for baseline weight. Subjects without follow up (dropouts) will have values imputed using multiple imputation. This approach assumes the distribution of missing data is random after conditioning on observed data in the imputation process. A secondary approach will consider complete case data, though this does not generally adhere to ITT principles.

Weight is recorded longitudinally at several post-baseline timepoints. A secondary analysis will model these longitudinal data using linear mixed effects models, adjusted for baseline weight. Additional adjustment variables will be included based on *a priori* determination by investigators that such variables may be associated with the dropout process. Thus, any dropout is assumed Missing At Random as a function of observed data. Time after randomization will be included as a discrete ordinal variable corresponding to the visit number. The primary comparison is a time by treatment group interaction, so that contrasts of the variables in the model will allow estimation of the treatment effect at each visit.

An interim analysis will be performed after 50% of subjects have completed their 3 month visit. This will allow the study team to review data quality and check assumptions related to the power calculation (including the standard deviation above). Investigators will remain blinded to group comparisons and while investigators have no intention of stopping the study early (regardless of interim results), the analysis at study completion will be conservatively adjusted using the O’Brien-Fleming boundary (two-sided significance level of 0.0492).

Secondary endpoints will be assessed similarly. Steps, calories, and hours using the app tracker will be collected by the device. These will be aggregated to a weekly total or daily average over the week-long period to smooth out day-to-day variation. Analyses will be performed using linear mixed effects models with these weekly total data, separately for each endpoint. Weight loss as recorded by the Bluetooth scale at home will be analyzed similarly, taking the average of assessments over the course of each week as the outcome [recall, the primary endpoint is weight loss measured in clinic at study visits]. Number of exercise sessions will be evaluated, also as a weekly total number of sessions, using generalized linear mixed effects models with the outcome analyzed as a Poisson count. The distribution of each outcome will be assessed and alternative approaches considered as necessary. Quality of Life questionnaires will be assessed at study visits and analyzed using linear mixed effects models to compare between groups. The primary outcome has been pre-specified and no adjustment will be made for comparisons of these secondary outcomes.

An exploratory aim will evaluate adherent vs non-adherent patients – and further the interaction between adherent (vs non-adherent) and randomized group, to assess whether there is a differential response to Phentermine-Topiramate among exercise adherent patients. Adherence will be defined by 80% use during the first 3 months.

# Anticipated results and significance:

Our study will demonstrate the importance of combining a consumer-based wearable activity tracker with an Anti-obesity Pharmacotherapy in Obesity.

# Potential pitfalls, precautions taken, and alternative strategies:

1. Feasibility - Given high volume of patients interested in weight loss, we are confident we will recruit sufficient participants for these studies that involve only noninvasive tests and standard of care treatment.
2. Statistical power has been addressed with appropriate sample sizes to demonstrate a difference in weight change vs. placebo.

# References

[1] Gadde KM, Allison DB, Ryan DH, Peterson CA, Troupin B, Schwiers ML, et al. Effects of low-dose, controlled-release, phentermine plus topiramate combination on weight and associated comorbidities in overweight and obese adults (CONQUER): a randomised, placebo-controlled, phase 3 trial. Lancet. 2011;377:1341-52.

[2] Ng M, Fleming T, Robinson M, Thomson B, Graetz N, Margono C, et al. Global, regional, and national prevalence of overweight and obesity in children and adults during 1980-2013: a systematic analysis for the Global Burden of Disease Study 2013. Lancet. 2014.

[3] Flegal KM, Carroll MD, Kit BK, Ogden CL. Prevalence of obesity and trends in the distribution of body mass index among US adults, 1999-2010. JAMA : the journal of the American Medical Association. 2012;307:491-7.

[4] Hensrud DD, Klein S. Extreme obesity: a new medical crisis in the United States. Mayo Clin Proc. 2006;81:S5-10.

[5] Yach D, Stuckler D, Brownell KD. Epidemiologic and economic consequences of the global epidemics of obesity and diabetes. Nat Med. 2006;12:62-6.

[6] Daniels SR, Jacobson MS, McCrindle BW, Eckel RH, Sanner BM. American Heart Association Childhood Obesity Research Summit: executive summary. Circulation. 2009;119:2114-23.

[7] Low S, Chin MC, Deurenberg-Yap M. Review on epidemic of obesity. Ann Acad Med Singapore. 2009;38:57-9.

[8] Ogden CL, Yanovski SZ, Carroll MD, Flegal KM. The epidemiology of obesity. Gastroenterology. 2007;132:2087-102.

[9] World-Health-Organisation. Fact sheet: obesity and overweight. . Available

at: <http://wwwwhoint/mediacentre/factsheets/fs311/en/printhtml2012>.

[10] Tsai AG, Williamson DF, Glick HA. Direct medical cost of overweight and obesity in the USA: a quantitative systematic review. Obes Rev. 2011;12:50-61.

[11] Riebe D, Franklin BA, Thompson PD, Garber CE, Whitfield GP, Magal M, et al. Updating ACSM's Recommendations for Exercise Preparticipation Health Screening. Med Sci Sports Exerc. 2015;47:2473-9.

[12] Jensen MD, Ryan DH, Apovian CM, Ard JD, Comuzzie AG, Donato KA, et al. 2013 AHA/ACC/TOS Guideline for the Management of Overweight and Obesity in Adults: A Report of the American College of Cardiology/American Heart Association Task Force on Practice Guidelines and The Obesity Society. J Am Coll Cardiol. 2014;63:2985-3023.

[13] Apovian CM, Aronne LJ, Bessesen DH, McDonnell ME, Murad MH, Pagotto U, et al. Pharmacological management of obesity: an endocrine Society clinical practice guideline. J Clin Endocrinol Metab. 2015;100:342-62.

[14] Sjostrom L, Rissanen A, Andersen T, Boldrin M, Golay A, Koppeschaar HP, et al. Randomised placebo-controlled trial of orlistat for weight loss and prevention of weight regain in obese patients. European Multicentre Orlistat Study Group. Lancet. 1998;352:167-72.

[15] Hollander PA, Elbein SC, Hirsch IB, Kelley D, McGill J, Taylor T, et al. Role of orlistat in the treatment of obese patients with type 2 diabetes. A 1-year randomized double-blind study. Diabetes Care. 1998;21:1288-94.

[16] Davidson MH, Hauptman J, DiGirolamo M, Foreyt JP, Halsted CH, Heber D, et al. Weight control and risk factor reduction in obese subjects treated for 2 years with orlistat: a randomized controlled trial. Jama. 1999;281:235-42.

[17] Gadde KM, Allison DB, Ryan DH, Peterson CA, Troupin B, Schwiers ML, et al. Effects of low-dose, controlled-release, phentermine plus topiramate combination on weight and associated comorbidities in overweight and obese adults (CONQUER): a randomised, placebo-controlled, phase 3 trial. Lancet. 2011;377:1341-52.

[18] Smith S, Weissman N, Anderson C, Sanchez M, Chuang E, Stubbe S, et al. Multicenter, placebo-controlled trial of lorcaserin for weight management. The New England journal of medicine. 2010;363:245-56.

[19] Apovian CM, Aronne L, Rubino D, Still C, Wyatt H, Burns C, et al. A randomized, phase 3 trial of naltrexone SR/bupropion SR on weight and obesity-related risk factors (COR-II). Obesity (Silver Spring). 2013;21:935-43.

[20] Pi-Sunyer X, Astrup A, Fujioka K, Greenway F, Halpern A, Krempf M, et al. A Randomized, Controlled Trial of 3.0 mg of Liraglutide in Weight Management. New Engl J Med. 2015;373:11-22.

[21] MD R. US Food and Drug Administration Endocrinologic and Metabolic Drugs Advisory Committee Clinical Briefing Document February 22, 2012. [Accessed: 3/30/2012];VIVUS, Inc New Drug Application 2 2 5 8 0 : V I-0521 QNEXA (phentermine/topiramate) Web February 22, 2012.

[22] Garvey WT, Ryan DH, Look M, Gadde KM, Allison DB, Peterson CA, et al. Two-year sustained weight loss and metabolic benefits with controlled-release phentermine/topiramate in obese and overweight adults (SEQUEL): a randomized, placebo-controlled, phase 3 extension study. Am J Clin Nutr. 2012;95:297-308.

[23] Allison DB, Gadde KM, Garvey WT, Peterson CA, Schwiers ML, Najarian T, et al. Controlled-release phentermine/topiramate in severely obese adults: a randomized controlled trial (EQUIP). Obesity (Silver Spring). 2012;20:330-42.

[24] Lyons EJ, Lewis ZH, Mayrsohn BG, Rowland JL. Behavior change techniques implemented in electronic lifestyle activity monitors: a systematic content analysis. J Med Internet Res. 2014;16:e192.

[25] Preusse KC, Mitzner TL, Fausset CB, Rogers WA. Older Adults' Acceptance of Activity Trackers. J Appl Gerontol. 2017;36:127-55.

[26] Cadmus-Bertram LA, Marcus BH, Patterson RE, Parker BA, Morey BL. Randomized Trial of a Fitbit-Based Physical Activity Intervention for Women. Am J Prev Med. 2015;49:414-8.

[27] Skrepnik N, Spitzer A, Altman R, Hoekstra J, Stewart J, Toselli R. Assessing the Impact of a Novel Smartphone Application Compared With Standard Follow-Up on Mobility of Patients With Knee Osteoarthritis Following Treatment With Hylan G-F 20: A Randomized Controlled Trial. JMIR Mhealth Uhealth. 2017;5:e64.

[28] Brickwood KJ, Watson G, O'Brien J, Williams AD. Consumer-Based Wearable Activity Trackers Increase Physical Activity Participation: Systematic Review and Meta-Analysis. JMIR Mhealth Uhealth. 2019;7:e11819.

[29] Vazquez Roque MI, Camilleri M, Stephens DA, Jensen MD, Burton DD, Baxter KL, et al. Gastric sensorimotor functions and hormone profile in normal weight, overweight, and obese people. Gastroenterology. 2006;131:1717-24.

[30] Acosta A, Camilleri M, Shin A, Vazquez-Roque MI, Iturrino J, Burton D, et al. Quantitative gastrointestinal and psychological traits associated with obesity and response to weight-loss therapy. Gastroenterology. 2015;148:537-46 e4.

[31] Camilleri M, Iturrino J, Bharucha A, Burton D, Shin A, Jeong ID, et al. Performance characteristics of scintigraphic measurement of gastric emptying of solids in healthy participants. Neurogastroenterology and motility : the official journal of the European Gastrointestinal Motility Society. 2012;24:1076.

[32] Bush K, Kivlahan DR, McDonell MB, Fihn SD, Bradley KA. The AUDIT alcohol consumption questions (AUDIT-C): an effective brief screening test for problem drinking. Ambulatory Care Quality Improvement Project (ACQUIP). Alcohol Use Disorders Identification Test. Arch Intern Med. 1998;158:1789-95.

[33] Yanovski SZ, Marcus MD, Wadden TA, Walsh BT. The Questionnaire on Eating and Weight Patterns-5: an updated screening instrument for binge eating disorder. Int J Eat Disord. 2015;48:259-61.

[34] Clark MM, Novotny PJ, Patten CA, Rausch SM, Garces YI, Jatoi A, et al. Motivational readiness for physical activity and quality of life in long-term lung cancer survivors. Lung cancer. 2008;61:117-22.

[35] Markland D, Tobin, V. A modification of the Behavioral Regulation in Exercise Questionnaire to include an assessment of amotivation. Journal of Sport and Exercise Psychology. 2004;26:191-6.

[36] Khemani D, Eckert DJ, O'Neill J, Ryks M, Rhoten D, Acosta Cardenas AJ, et al. Effect of Liraglutide on Gastric Emptying at 5 and 16 Weeks: A Single-Center, Randomized, Placebo-Controlled Trial in 32 Patients. Gastroenterology.152:S83.


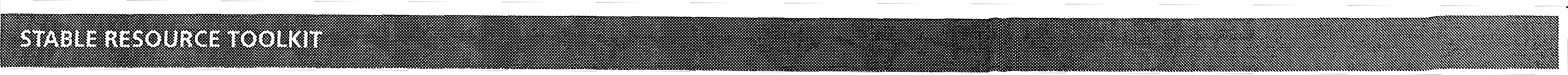


AUDIT-C Questionnaire

Date of Visit _ _/_ _/_ _ _ _

Subject Number

MM DD YYYY

# How often do you have a drink containing alcohol?

D a. Never

D b. Monthly or less D c. 2-4 times a month D d. 2-3 times a week

D e. 4 or more times a week

# How many standard drinks containing alcohol do you have on a typical day?

□

a. 1 or 2

□

1. 3 or 4

□

1. 5 or 6

□

1. 7 to 9

□

1. 10 or more

# How often do you have six or more drinks on one occasion?

D a. Never

D b. Less than monthly

- - c. Monthly
  - d. Weekly

D e. Daily or almost daily

*SIGN:*

*AUDIT-C is available for use in the public domain.*

*DATE:* */*  */*

*MM DD Y*

# Study ID:_ _ _:_ _ _

## EXERCISE REGULATIONS QUESTIONNAIRE (BREQ-3)

Number Initials

# Date:_ _/_ _/_ _ _ _

## Age: years Sex: male female (please circle)

***WHY DO YOU ENGAGE IN EXERCISE?***

MM DD YYYY

We are interested in the reasons underlying peoples’ decisions to engage or not engage in physical exercise. Using the scale below, please indicate to what extent each of the following items is true for you. Please note that there are no right or wrong answers and no trick questions. We simply want to know how you personally feel about exercise. Your responses will be held in confidence and only used for our research purposes.

|  | **Not true for me** |  | **Sometimes true for me** |  | **Very true for me** |
| --- | --- | --- | --- | --- | --- |
| 1 It’s important to me to exercise regularly | 0 | 1 | 2 | 3 | 4 |
| 2 I don’t see why I should have to exercise | 0 | 1 | 2 | 3 | 4 |
| 3 I exercise because it’s fun | 0 | 1 | 2 | 3 | 4 |
| 4 I feel guilty when I don’t exercise | 0 | 1 | 2 | 3 | 4 |
| 5 I exercise because it is consistent with my life goals | 0 | 1 | 2 | 3 | 4 |
| 6 I exercise because other people say I should | 0 | 1 | 2 | 3 | 4 |
| 7 I value the benefits of exercise | 0 | 1 | 2 | 3 | 4 |
| 8 I can’t see why I should bother exercising | 0 | 1 | 2 | 3 | 4 |
| 9 I enjoy my exercise sessions | 0 | 1 | 2 | 3 | 4 |
| 10 I feel ashamed when I miss an exercise session | 0 | 1 | 2 | 3 | 4 |
| 11 I consider exercise part of my identity | 0 | 1 | 2 | 3 | 4 |
| 12 I take part in exercise because my friends/family/partner say I should | 0 | 1 | 2 | 3 | 4 |
| 13 I think it is important to make the effort to exercise regularly | 0 | 1 | 2 | 3 | 4 |
| 14 I don’t see the point in exercising | 0 | 1 | 2 | 3 | 4 |
| 15 I find exercise a pleasurable activity | 0 | 1 | 2 | 3 | 4 |
| 16 I feel like a failure when I haven’t exercised in a while | 0 | 1 | 2 | 3 | 4 |
| 17 I consider exercise a fundamental part of who I am | 0 | 1 | 2 | 3 | 4 |
| 18 I exercise because others will not be pleased with me if I don’t | 0 | 1 | 2 | 3 | 4 |
| 19 I get restless if I don’t exercise regularly | 0 | 1 | 2 | 3 | 4 |
| 20 I think exercising is a waste of time | 0 | 1 | 2 | 3 | 4 |

|  | **Not true for me** |  | **Sometimes true for me** |  | **Very true for me** |
| --- | --- | --- | --- | --- | --- |
| 21 I get pleasure and satisfaction from participating in exercise | 0 | 1 | 2 | 3 | 4 |
| 22 I would feel bad about myself if I was not making time to exercise | 0 | 1 | 2 | 3 | 4 |
| 23 I consider exercise consistent with my values | 0 | 1 | 2 | 3 | 4 |
| 24 I feel under pressure from my friends/family to exercise | 0 | 1 | 2 | 3 | 4 |


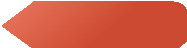


## Thank you for taking part in our research

David Markland PhD, C.Psychol

School of Sport, Health & Exercise Sciences University of Wales, Bangor [d.a.markland@bangor.ac.uk](mailto:d.a.markland@bangor.ac.uk)

October 2014

**Eating Patterns Questionnaire**

Subject Initials_________

Screen Number________

| **__** 15 | 1. During the past **six** months, did you often eat within any two-hour period what most people would regard as an unusually large amount of food?   1 Yes 0 No → SKIP TO QUESTION 5 | |
| --- | --- | --- |
| **__** 16 | 1. During the times when you ate this way, did you often feel you couldn’t stop eating or control what or how much you were eating?   1 Yes 0 No → SKIP TO QUESTION 5 | |
|  | 1. During the past **six** months, how often, on average, did you have times when you ate this way – that is, large amounts of food **plus** the feeling that your eating was out of control (there may have been some weeks when it was not present – just average those in). | |
| **__** 17 | 1 Less than one day a week  2 One day a week  3 Two or three days a week | 4 Four to five times a week  5 Nearly every day |
|  | 4. Did you usually have any of the following experiences during these occasions? | |
| **__** 18 | a. Eating much more rapidly than usual? 1 Yes 0 No | |
| **__** 19 | b. Eating until you felt uncomfortably full? 1 Yes 0 No | |
| **__** 20 | c. Eating large amounts of food when you didn’t  feel physically hungry? 1 Yes 0 No | |
| **__** 21 | d. Eating alone because you were embarrassed  by how much you were eating? 1 Yes 0 No | |
| **__** 22 | e. Feeling disgusted with yourself, depressed,  or feeling very guilty after overeating? 1 Yes 0 No | |
|  | 5. In general, during the past **six** months, how upset were you by overeating (*eating more than you think is best for you*)? | |
| **__** 23 | 1 Not at all  2 Slightly  3 Moderately | 4 Greatly  5 Extremely |
|  | 6. In general, during the past **six** months, how upset were you by the feeling that you couldn’t stop eating or control what or how much you were eating? | |
| **__** 24 | 1 Not at all  2 Slightly  3 Moderately | 4 Greatly  5 Extremely |

|  | 7. During the past six months, how important has your weight or shape been, in how you feel about or evaluate yourself as a person – as compared to other aspects of your life, such as how you do at work as a parent, or how you get along with other people? | | |
| --- | --- | --- | --- |
| **__** 25 | 1 Weight and shape were **not very important**  2 Weight and shape **played a part** in how you felt about yourself  3 Weight and shape **were among the main things** that affected how you felt about yourself  4 Weight and shape **were the most important things** that affected how you felt about yourself | | |
|  | 8. During the past **three** months, did you ever make yourself vomit in order to avoid gaining weight after binge eating? | | |
| __ 26  __ 27 | 1 Yes ⎯⎯→  0 No | How often, **on average**, was that?  1 Less than one day a week  2 One day a week  3 Two or three days a week  4 Four to five times a week  5 More than five times a week |  |
|  |  | | |
|  | 9. During the past **three** months, did you ever take more than twice the recommended dose of laxatives in order to avoid gaining weight after binge eating? | | |
| __ 27  __ 28 | 1 Yes ⎯⎯→  0 No | How often, **on average**, was that?  1 Less than one day a week  2 One day a week  3 Two or three days a week  4 Four to five times a week  5 More than five times a week |  |
|  |  | | |
|  | 10. During the past three months, did you ever take more than twice the recommended dose of diuretics (water pills) in order to avoid gaining weight after binge eating? | | |
| __ 29  __ 30 | 1 Yes ⎯⎯→  0 No | How often, **on average**, was that?  1 Less than one day a week  2 One day a week  3 Two or three days a week  4 Four to five times a week  5 More than five times a week |  |
|  |  | | |

|  | 11. During the past three months, did you ever fast – not eat anything at all for at least 24 hours --in order to avoid gaining weight after binge eating? | | |
| --- | --- | --- | --- |
| __ 31  __ 32 | 1 Yes ⎯⎯→  0 No | How often, **on average**, was that?  1 Less than one day a week  2 One day a week  3 Two or three days a week  4 Four to five times a week  5 Nearly every day |  |
|  |  | | |
|  | 12. During the past three months, did you ever exercise for more than an hour **specifically** in order to avoid gaining weight after binge eating? | | |
| __ 33  __ 34 | 1 Yes ⎯⎯→  0 No | How often, **on average**, was that?  1 Less than one day a week  2 One day a week  3 Two or three days a week  4 Four to five times a week  5 More than five times a week |  |
|  |  | | |
|  | 13. During the past three months, did you ever take more than twice the recommended dose of a diet pill in order to avoid gaining weight after binge eating? | | |
| __ 35  __ 36 | 1 Yes ⎯⎯→  0 No | How often, **on average**, was that?  1 Less than one day a week  2 One day a week  3 Two or three days a week  4 Four to five times a week  5 More than five times a week |  |

# Sign____________________________________ Date _________________

## Subjects Initials ______

# Screen Number ______

# The Hospital Anxiety and Depression Questionnaire

Please read each item and **circle** the reply which best describes how you have been feeling during the past week. Don’t devote too much time to your responses; your immediate reaction will probably be more accurate than a long thought out response.

1. I feel tense or ‘wound up’ :

Most of the time

A lot of the time

Occasionally

Not at all

1. I still enjoy the things I used to enjoy :

Definitely as much

Not quite so much

Only a little

Hardly at all

1. I get a frightened feeling, as if something awful is about to happen :

Very definitely and quite badly

Yes, but not too badly

A little, but it doesn’t worry me

Not at all

1. I can laugh and see the funny side of things :

As much as I always could

Not quite so much now

Definitely not so much now

Not at all

1. Worrying thoughts go through my mind :

A great deal of the time

A lot of the time

From time to time

Only occasionally

1. I feel cheerful :

Not at all

Not often

Sometimes

Most of the time

1. I can sit at ease and feel relaxed :

Definitely

Usually

Not often

Not at all

1. I feel as if I am slowed down :

Nearly all the time

Very often

Sometimes

Not at all

1. I get a frightened feeling, like ‘butterflies in the stomach’ :

Not at all

Occasionally

Quite often

Very often

1. I have lost interest in my appearance :

Definitely

I don’t take as much care as I should

I may not take quite as much care

I take just as much care as ever

1. I feel restless as if I have to be on the move :

Very much indeed

Quite a lot

Not very much

Not at all

1. I look forward with enjoyment to things :

As much as I ever did

Rather less than I used to

Definitely less than I used to

Hardly at all

1. I get sudden feelings of panic :

Very often indeed

Quite often

Not very often

Not at all

1. I can enjoy a good book or TV program :

Often

Sometimes

Not often

Very seldom

Visual Analog Scale

### Sign___________________________________ Date _______________

**0 Minute**

# Directions: Answer each question about how you are feeling by drawing a vertical mark (|) at the appropriate point through the horizontal line of each question.

How hungry do you feel?

**I have never**

**been more hungry**

**I am not**

**hungry at all**

How satisfied do you feel?

**I am**

**completely**

**empty**

**I cannot eat**

**another bite**

How full do you feel?

**Not at all full**

**Totally full**

How much do you think you can eat?

**A lot**

**Nothing at all**

Would you like to eat something sweet?

**Yes, very much**

**No, not at all**

Would you like to eat something salty?

**No, not at all**

**Yes, very much**

Would you like to eat something savoury?

**No, not at all**

**Yes, very much**

Would you like to eat something fatty?

**Yes, very much**

**No, not at all**

**30 Minute**

# Directions: Answer each question about how you are feeling by drawing a vertical mark (|) at the appropriate point through the horizontal line of each question.

How hungry do you feel?

**I have never**

**been more hungry**

**I am not**

**hungry at all**

How satisfied do you feel?

**I am**

**completely**

**empty**

**I cannot eat**

**another bite**

How full do you feel?

**Not at all full**

**Totally full**

How much do you think you can eat?

**A lot**

**Nothing at all**

Would you like to eat something sweet?

**Yes, very much**

**No, not at all**

Would you like to eat something salty?

**No, not at all**

**Yes, very much**

Would you like to eat something savoury?

**No, not at all**

**Yes, very much**

Would you like to eat something fatty?

**Yes, very much**

**No, not at all**

**60 Minute**

# Directions: Answer each question about how you are feeling by drawing a vertical mark (|) at the appropriate point through the horizontal line of each question.

How hungry do you feel?

**I have never**

**been more hungry**

**I am not**

**hungry at all**

How satisfied do you feel?

**I am**

**completely**

**empty**

**I cannot eat**

**another bite**

How full do you feel?

**Not at all full**

**Totally full**

How much do you think you can eat?

**A lot**

**Nothing at all**

Would you like to eat something sweet?

**Yes, very much**

**No, not at all**

Would you like to eat something salty?

**No, not at all**

**Yes, very much**

Would you like to eat something savoury?

**No, not at all**

**Yes, very much**

Would you like to eat something fatty?

**Yes, very much**

**No, not at all**

**90 Minute**

# Directions: Answer each question about how you are feeling by drawing a vertical mark (|) at the appropriate point through the horizontal line of each question.

How hungry do you feel?

**I have never**

**been more hungry**

**I am not**

**hungry at all**

How satisfied do you feel?

**I am**

**completely**

**empty**

**I cannot eat**

**another bite**

How full do you feel?

**Not at all full**

**Totally full**

How much do you think you can eat?

**A lot**

**Nothing at all**

Would you like to eat something sweet?

**Yes, very much**

**No, not at all**

Would you like to eat something salty?

**No, not at all**

**Yes, very much**

Would you like to eat something savoury?

**No, not at all**

**Yes, very much**

Would you like to eat something fatty?

**Yes, very much**

**No, not at all**

**120 Minute**

# Directions: Answer each question about how you are feeling by drawing a vertical mark (|) at the appropriate point through the horizontal line of each question.

How hungry do you feel?

**I have never**

**been more hungry**

**I am not**

**hungry at all**

How satisfied do you feel?

**I am**

**completely**

**empty**

**I cannot eat**

**another bite**

How full do you feel?

**Not at all full**

**Totally full**

How much do you think you can eat?

**A lot**

**Nothing at all**

Would you like to eat something sweet?

**Yes, very much**

**No, not at all**

Would you like to eat something salty?

**No, not at all**

**Yes, very much**

Would you like to eat something savoury?

**No, not at all**

**Yes, very much**

Would you like to eat something fatty?

**Yes, very much**

**No, not at all**

# Physical Activity Stages of Change Questionnaire

Subject Initials_________

Screen Number________

For each of the questions below, please check Yes or No. Please be sure to follow the instructions carefully.

Physical activity or exercise includes activities such as walking briskly, jogging, bicycling, swimming or any other activity where the exertion is as least as hard as these activities. Your heart rate and breathing should increase.

|  | NO | YES |
| --- | --- | --- |
| 1. I am currently **physically active** |  |  |
| 2. I intend to become more **physically active** in the next 6 months |  |  |

For activity to be **regular**, it must add up to a **total** of **30 minutes or more per day**, and be done **at least 5 days per week**. For example, you could take one 30 minute walk, or take three 10 minute walks each day.

|  | NO | YES |
| --- | --- | --- |
| 3. I currently engage in **regular physical activity** |  |  |
| 4. I have been **regularly physically active** for the past 6 months |  |  |

How many days, over the past week, have you engaged in at least 30 minutes of physical activity? ____________

# Sign_________________________________ Date _______________

Study ID:_ _ _:_ _ _

Number Initials

Date:_ _/_ _/_ _ _ _

MM DD YYYY

Your Health and Well-Being

This survey asks for your views about your health. This information will help keep track of how you feel and how well you are able to do your usual activities. *Thank you for completing this survey!*

For each of the following questions, please mark an
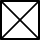
 in the one box that best describes your answer.

1. **In general, would you say your health is:**


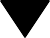

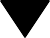

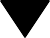

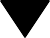

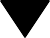


Excellent Very good Good Fair Poor

1 2 3 4 5

1. **The following questions are about activities you might do during a typical day. Does your health now limit you in these activities? If so, how**

much?


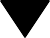

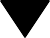

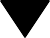


Yes, limited a lot

Yes, limited a little

No, not limited at all

a Moderate activities, such as moving a table,

| pushing a vacuum cleaner, bowling, or |  | | |
| --- | --- | --- | --- |
| playing golf........................................................................ | 1 ............ | 2............ | 3 |
| b Climbing several flights of stairs ........................................ | 1 ............ | 2............ | 3 |

1. **During the past 4 weeks, how much of the time have you had any of the follow result of your physical health?**


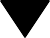

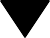

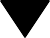

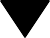

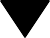


All of Most of Some A little None the time the time of the of the of the

time time time

a Accomplished less than you would

like ..................................................................

b Were limited in the kind of work or

other activities...................................................

1....... 2 ........ 3 ....... 4 5

1....... 2 ........ 3 ....... 4 5

1. **During the past 4 weeks, how much of the time have you had any of the following problems with your work or other regular daily activities as a result of any emotional problems (such as feeling depressed or anxious)?**


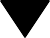

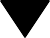

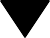

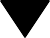

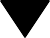


All of Most of Some

the time the time of the

time

A little None

of the of the

time time

a Accomplished less than you would like .............

1........

2 .......

3 .......

4 5

b Did work or other activities less

carefully than usual...........................................

1........ 2 ....... 3 ....... 4 5

1. **During the past 4 weeks, how much did pain interfere with your normal work (including both work outside the home and housework)?**


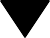

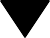

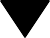

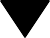

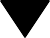


Not at all A little bit Moderately Quite a bit Extremely

1 2 3 4 5

1. **These questions are about how you feel and how things have been with you during the past 4 weeks. For each question, please give the one answer that comes closest to the way you have been feeling. How much of the time during the past 4 weeks...**


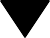

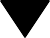

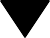

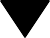

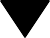


All

of

the time

Most of the time

Some of A little of None of

the time

the time

the time

a Have you felt calm and peaceful? ................

| 1 ......... | 2 .......... | 3 .......... | 4 .......... | 5 |
| --- | --- | --- | --- | --- |
| 1 ......... | 2 .......... | 3 .......... | 4 .......... | 5 |
| 1 ......... | 2 .......... | 3 .......... | 4 .......... | 5 |

b Did you have a lot of energy?......................

c Have you felt downhearted and depressed?.................................................

1. **During the past 4 weeks, how much of the time has your physical health or emotional problems interfered with your social activities (like visiting friends, relatives, etc.)?**


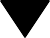

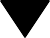

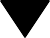

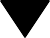

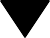


All of the time

Most of the time

Some of the time

A little of the time

None of the time

1 2 3 4 5

***Thank you for completing these questions!***

Sign Date

Study ID:_ _ _:_ _ _

Number Initials

Date:_ _/_ _/_ _ _ _

MM DD YYYY

STOP-BANG Sleep Apnea Questionnaire

***Chung F et al Anesthesiology 2008 and BJA 2012***

| **STOP** |  |  |
| --- | --- | --- |
| Do you **S**NORE loudly (louder than talking or loud enough to be heard through closed doors)? | Yes | No |
| Do you often feel **T**IRED, fatigued, or sleepy during daytime? | Yes | No |
| Has anyone **O**BSERVED you stop breathing during your sleep? | Yes | No |
| Do you have or are you being treated for high blood  **P**RESSURE? | Yes | No |

| **BANG** |  |  |
| --- | --- | --- |
| **B**MI more than 35kg/m2? | Yes | No |
| **A**GE over 50 years old? | Yes | No |
| **N**ECK circumference > 16 inches (40cm)? | Yes | No |
| **G**ENDER: Male? | Yes | No |

| **TOTAL SCORE** |  |  |
| --- | --- | --- |

High risk of OSA: Yes 5 - 8 Intermediate risk of OSA: Yes 3 - 4 Low risk of OSA: Yes 0 - 2

Sign Date

Subject Number: _________________________ Date: __________________

**Three-Factor Eating Questionnaire-R21**

*International Journal of Obesity (2009) 33, 611–620*

The Three-Factor Eating Questionnaire—Revised 21-Item (TFEQ-R21)

**Please answer the following question to the best of your ability, and as they relate to your current state.**

**1. I deliberately take small helpings to control my weight.**

(1) Definitely true, (2) Mostly true, (3) Mostly false, (4) Definitely false

**2. I start to eat when I feel anxious.**

(1) Definitely true, (2) Mostly true, (3) Mostly false, (4) Definitely false

**3. Sometimes when I start eating, I just can't seem to stop.**

(1) Definitely true, (2) Mostly true, (3) Mostly false, (4) Definitely false

**4. When I feel sad, I often eat too much.**

(1) Definitely true, (2) Mostly true, (3) Mostly false, (4) Definitely false

**5. I don't eat some foods because they make me fat.**

(1) Definitely true, (2) Mostly true, (3) Mostly false, (4) Definitely false

**6. Being with someone who is eating, often makes me want to also eat.**

(1) Definitely true, (2) Mostly true, (3) Mostly false, (4) Definitely false

**7. When I feel tense or "wound up", I often feel I need to eat.**

(1) Definitely true, (2) Mostly true, (3) Mostly false, (4) Definitely false

**8. I often get so hungry that my stomach feels like a bottomless pit.**

(1) Definitely true, (2) Mostly true, (3) Mostly false, (4) Definitely false

**9. I'm always so hungry that it's hard for me to stop eating before finishing all of the food on my plate.**

(1) Definitely true, (2) Mostly true, (3) Mostly false, (4) Definitely false

**10. When I feel lonely, I console myself by eating.**

(1) Definitely true, (2) Mostly true, (3) Mostly false, (4) Definitely false

**11. I consciously hold back on how much I eat at meals to keep from gaining weight.**

(1) Definitely true, (2) Mostly true, (3) Mostly false, (4) Definitely false

**12. When I smell a sizzling steak or see a juicy piece of meat, I find it very difficult to keep from eating—even if I've just finished a meal.**

(1) Definitely true, (2) Mostly true, (3) Mostly false, (4) Definitely false

**13. I'm always hungry enough to eat at any time.**

(1) Definitely true, (2) Mostly true, (3) Mostly false, (4) Definitely false

**14. If I feel nervous, I try to calm down by eating.**

(1) Definitely true, (2) Mostly true, (3) Mostly false, (4) Definitely false

**15. When I see something that looks very delicious, I often get so hungry that I have to eat right away.**

(1) Definitely true, (2) Mostly true, (3) Mostly false, (4) Definitely false

**16. When I feel depressed, I want to eat.**

(1) Definitely true, (2) Mostly true, (3) Mostly false, (4) Definitely false

**17. How often do you avoid "stocking up" on tempting foods?**

(1) Almost never, (2) Seldom, (3) Usually, (4) Almost always

**18. How likely are you to make an effort to eat less than you want?**

(1) Unlikely, (2) A little likely, (3) Somewhat likely, (4) Very likely.

**19. Do you go on eating binges even though you're not hungry?**

(1) Never, (2) Rarely, (3) Sometimes, (4) At least once a week

**20. How often do you feel hungry?**

(1) Only at mealtimes, (2) Sometimes between meals (3) Often between meals (4) Almost always

**21. On a scale from 1 to 8, where 1 means no restraint in eating and 8 means total restraint, what number would you give yourself?** Mark the number that best applies to you: 1 2 3 4 5 6 7

# Study ID:_ _ _:_ _ _

Number Initials

# Date:_ _/_ _/_ _ _ _

WOMAC OSTEOARTHRITIS INDEX

MM DD YYYY

1. **The following questions concern the amount of pain you are currently experiencing in your knees. For each situation, please enter the amount of pain you have experienced in the past 48 hours.**

| **A. Walking on a flat surface** | **None**  **A.** | **mild moderate severe** | **extreme** |
| --- | --- | --- | --- |
| **B. Going up or down stairs** | **B.** |  |  |
| 1. **At night while in bed** 2. **Sitting or lying** | **C.**  **D.** |  |  |
| **E. Standing upright** | **E.** |  |  |

1. **Please describe the level of pain you have experienced in the past 48 hours for each one of your knees.**

None mild moderate severe extreme

- 1. **Right knee A.**
  2. **Left knee B.**

1. **How severe is your stiffness after first awakening in the morning?**

None mild moderate severe extreme

1. **How severe is your stiffness after sitting, lying, or resting later in the day?**

None mild moderate severe extreme

1. **The following questions concern your physical function. By this we mean your ability to move around and to look after yourself. For each of the following activities, please indicate the degree of difficulty you have experienced in the last 48 hours, in your knees.**

What degree of difficulty do you have with:

- 1. **Descending (going down) stairs A.**
  2. **Ascending (going up) stairs B.**
  3. **Rising from sitting C.**
  4. **Standing D.**
  5. **Bending to floor E.**
  6. **Walking on a flat surface F.**
  7. **Getting in/out of car G.**
  8. **Going shopping H.**
  9. **Putting on socks/stockings I.**
  10. **Rising from bed J.**
  11. **Taking off socks/stockings K.**
  12. **Lying in bed L.**
  13. **Getting in/out of bath M.**
  14. **Sitting N.**
  15. **Getting on/off toile O.**
  16. **Heavy domestic duties (mowing P. the lawn, lifting heavy grocery bags)**
  17. **Light domestic duties (such as Q. tidying a room, dusting, cooking)**

None mild moderate severe extreme

Sign Date
